# Functionalized Metallic 2D Transition Metal Dichalcogenide-Based Solid-State Electrolyte for Flexible All-Solid-State Supercapacitors

**DOI:** 10.1021/acsnano.2c05640

**Published:** 2022-10-04

**Authors:** Ahmad Bagheri, Sebastiano Bellani, Hossein Beydaghi, Matilde Eredia, Leyla Najafi, Gabriele Bianca, Marilena Isabella Zappia, Milad Safarpour, Maedeh Najafi, Elisa Mantero, Zdenek Sofer, Guorong Hou, Vittorio Pellegrini, Xinliang Feng, Francesco Bonaccorso

**Affiliations:** † Graphene Labs, Istituto Italiano di Tecnologia, via Morego 30, 16163 Genoa, Italy; ‡ Center for Advancing Electronics Dresden (CFAED) & Faculty of Chemistry and Food Chemistry, Technische Universität Dresden, 01062 Dresden, Germany; § BeDimensional SpA, Lungotorrente Secca 30R, 16163 Genoa, Italy; ∥ Dipartimento di Chimica e Chimica Industriale, Università degli Studi di Genova, via Dodecaneso 31, 16146 Genoa, Italy; ⊥ Smart Materials, Istituto Italiano di Tecnologia, Via Morego 30, 16163 Genova, Italy; # Dipartimento di Informatica Bioingegneria, Robotica e Ingegneria dei Sistemi (DIBRIS), Universita Degli Studi di Genova, Via All’Opera Pia 13, 16145 Genova, Italy; ∇ Department of Inorganic Chemistry, University of Chemistry and Technology Prague, Technicka 5, 166 28 Prague 6, Czech Republic; ⊗ Max Planck Institute of Microstructure Physics, Weinberg 2, 06120 Halle, Germany

**Keywords:** solid-state supercapacitors, transition metal dichalcogenides, niobium disulfide, functionalization, flexibility

## Abstract

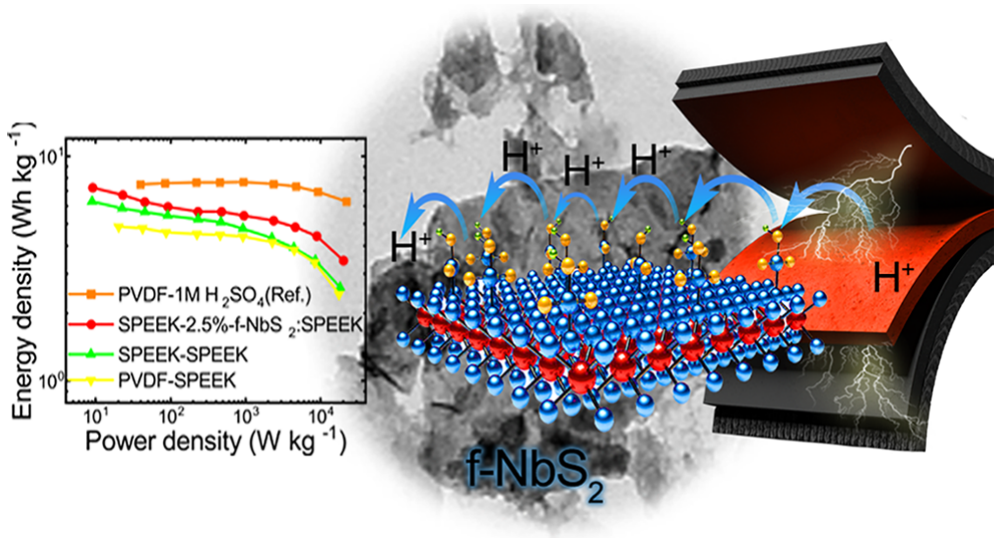

Highly efficient and durable flexible solid-state supercapacitors (FSSSCs) are emerging as low-cost devices for portable and wearable electronics due to the elimination of leakage of toxic/corrosive liquid electrolytes and their capability to withstand elevated mechanical stresses. Nevertheless, the spread of FSSSCs requires the development of durable and highly conductive solid-state electrolytes, whose electrochemical characteristics must be competitive with those of traditional liquid electrolytes. Here, we propose an innovative composite solid-state electrolyte prepared by incorporating metallic two-dimensional group-5 transition metal dichalcogenides, namely, liquid-phase exfoliated functionalized niobium disulfide (f-NbS_2_) nanoflakes, into a sulfonated poly(ether ether ketone) (SPEEK) polymeric matrix. The terminal sulfonate groups in f-NbS_2_ nanoflakes interact with the sulfonic acid groups of SPEEK by forming a robust hydrogen bonding network. Consequently, the composite solid-state electrolyte is mechanically/dimensionally stable even at a degree of sulfonation of SPEEK as high as 70.2%. At this degree of sulfonation, the mechanical strength is 38.3 MPa, and thanks to an efficient proton transport through the Grotthuss mechanism, the proton conductivity is as high as 94.4 mS cm^–1^ at room temperature. To elucidate the importance of the interaction between the electrode materials (including active materials and binders) and the solid-state electrolyte, solid-state supercapacitors were produced using SPEEK and poly(vinylidene fluoride) as proton conducting and nonconducting binders, respectively. The use of our solid-state electrolyte in combination with proton-conducting SPEEK binder and carbonaceous electrode materials (mixture of activated carbon, single/few-layer graphene, and carbon black) results in a solid-state supercapacitor with a specific capacitance of 116 F g^–1^ at 0.02 A g^–1^, optimal rate capability (76 F g^–1^ at 10 A g^–1^), and electrochemical stability during galvanostatic charge/discharge cycling and folding/bending stresses.

## 1 Introduction

The global demand for energy and noticeable depletion of fossil fuels during the past decade has caused energy crises and environmental concerns,^1−3^ calling for a pathway toward the transformation of the global energy sector from fossil-based to zero-carbon, i.e., the so-called energy transition.^4,5^ In this context, energy storage technologies represent essential enablers of the energy transition, since they can overcome the issues related to the variable output of renewable energy sources,^6,7^ resulting in resilient decarbonized electric grids.^8,9^ Meanwhile, they also play a crucial role in developing decentralized power networks, i.e., the so-called “microgrids”, for small-scale self-sufficient organizations^10,11^ and even portable and wearable electronics.^12−15^

In this scenario, electrochemical double layer capacitors (EDLCs) represent a type of supercapacitors that have attracted considerable attention because of their high power density (>10 kW kg^–1^)^16^ and excellent electrochemical stability over hundreds of thousands of charge–discharge cycles,^17−19^ complementing the characteristics of high-capacity energy storage systems, e.g., lithium-ion batteries,^20,21^ or other energy storage units, including electrochemical (e.g., flow batteries, pseudocapacitors),^22,23^ chemical (e.g., power-to-gas-to-power),^24^ thermal (e.g., molten salt technology),^25^ and mechanical (e.g., pumped hydroelectric storage)^26^ ones. Among supercapacitors, EDLCs exclusively rely on nonfaradaic charge storage, namely the ion adsorption and the swapping of co-ions for counterions at electrode–electrolyte interfaces, determining the double layer capacitance.^27−29^ To further extend the applications of supercapacitors, flexible solid-state supercapacitors (FSSSCs) have attracted significant interest because of their distinctive mechanical properties (e.g., bendability and foldability),^30,31^ lightness and safety (absence of leakage of toxic and corrosive electrolytes),^32,33^ which, ideally, can be coupled with the main features of traditional EDLCs (e.g., high power density and long-term operation).^34−36^ These properties turn FSSSCs into suitable candidates for portable and wearable electronics, including biomedical implants and health monitoring devices.^37−40^

Generally, the performance metrics of FSSSCs, including specific energy/power densities and mechanical/electrochemical stabilities, depend on several factors, e.g., electrolyte^41,42^ and electrode materials.^43−46^ In particular, the electrolyte is a crucial component that not only separates the two electrodes composing the FSSSCs, but also provides the ion-conducting medium that transfers and balances the charges between two electrodes, on whose surface the electrical double layer is formed.^47,48^ Generally, solid-state electrolytes include both gel electrolytes (which, technically, are classified as quasi-solid state electrolytes^16,49^) and solid polymer electrolytes (SPEs).^49^ Compared to gel ones, SPEs typically show superior dimensional stability and mechanical strength.^50,51^ Examples of SPEs are proton-conducting polymers, such as Nafion (brand name for sulfonated tetrafluoroethylene based fluoropolymer-copolymer)^52^ and sulfonated poly(ether ether ketone) (SPEEK),^53,54^ which are also widely exploited in the form of proton-exchange membranes (PEMs) for several energy storage and conversion applications.^55,56^ Despite its high proton conductivity (σ, around 90 mS cm^–1^ at 25 °C) and its satisfactory mechanical and thermal stabilities,^4,57^ Nafion has a high cost (∼$200 USD, 30 × 30 cm^–2^ for Nafion 117)^58^ that may limit its application in practical FSSSCs, whose market uptake is still at its infancy. Alternatively, SPEEK is a hydrocarbon-based thermoplastic polymer with mechanical strength (tensile strength ∼37 MPa, i.e., 80% higher than Nafion 117),^59^ thermal stability (up to 300 °C),^60,61^ commercial availability of the polymeric precursor (i.e., poly(ether ether ketone) -PEEK-),^62^ and σ (up to 40 mS cm^–1^ at 25 °C) adequate for the massive development of solid-state electrolytes for FSSSCs.^54,63^ Furthermore, SPEEK has been extensively established as PEM material for fuel cells,^64−66^ electrolyzers,^67,68^ and redox-flow batteries.^69−72^ Importantly, the physical/electrical/(electro)chemical properties of SPEEK, including σ, water uptake (WU), and membrane swelling (MS), are determined by its degree of sulfonation (DS).^73,74^ More in detail, the presence of acidic functional groups, i.e., −HSO_3_^–^, attached to the hydrophobic backbone of the polymer, form hydrophilic domains that have ion transferring capabilities.^75^ Although σ increases with increasing DS, excessive sulfonation deteriorates the mechanical strength of SPEEK. To solve the dichotomy of SPEEK properties, the addition of proper fillers, such as metal oxides,^76^ perovskite nanoparticles^77^ and two-dimensional (2D) materials,^64,65,78,79^ represents a widespread strategy to mechanically reinforce high-DS SPEEK. Thanks to their high surface area, scalable production through liquid-phase exfoliation (LPE) methods,^80,81^ and facile functionalization through thermal, chemical, and physical treatments, 2D materials represent a broad class of additives for polymer reinforcement and functionality addition.^82^ Beyond graphene and its derivatives, transition metal dichalcogenides (TMDs), such as group-6 ones (i.e., MX_2_, in which M = Mo or W and X = S, Se, or Te) have been incorporated into proton-conducting polymers for the development of advanced nanocomposites. In fact, the hydrogen bonds between the sulfonate groups of SPEEK and the chalcogen terminations/functional groups of TMDs can improve the thermal, mechanical, chemical, electrical, and dimensional stabilities of the resulting nanocomposites.^83^ Meanwhile, they regulate the aggregation/separation of their hydrophilic/hydrophobic domains, affecting the proton transfer ability of the nanocomposites. Importantly, experimental studies have shown that functional groups can be easily introduced onto metallic defects (e.g., edge sites in the 2H phase of MoS_2_) and/or polar sites via covalent attachment or van der Waals bonds.^84−86^ Upon the incorporation of functionalized TMDs into polymeric matrixes, the abundance of functional groups (e.g., −SO_3_ ones) can then improve the proton transport properties of the pristine polymers. Importantly, the metallicity of the TMDs plays a primary role in the electron transfer between TMDs and reactant precursors, promoting efficient functionalization processes.^87^ Based on this rationale, 2D metallic group-5 TMDs, e.g., the tantalum disulfide (TaS_2_) nanoflakes, have been recently proposed as an ideal filler for the design of SPEEK-based composite PEMs thanks to their facile functionalization.^66^ Indeed, once sulfonated, TMDs expose −SO_3_ groups with a dual functional role: (1) mechanical reinforcement of SPEEK by establishing a robust hydrogen bonding network; (2) σ booster by participating in proton transferring mechanisms (either vehicle^79^ or Grotthuss mechanisms^88,89^).

Beyond the intrinsic properties of the solid-state electrolyte, the performance of FSSSC is strongly determined by the electrical connection between electrode materials and the electrolyte.^90−93^ In this context, the binders, used in the electrode material formulation to produce mechanically robust electrodes, must guarantee the ion transport from the solid-state electrolyte to electrode active materials for an effective electrical double layer formation.^90−93^ Consequently, ion-conducting binders may be ideal candidates since they intrinsically extend the ion-conducting pathways of the electrolyte in the proximity of the bound active materials,^91,92,94^ promoting high electrical double layer capacitance. Therefore, both Nafion^93^ and SPEEK^95^ have been proposed as potential ion-conducting binders for FSSSCs alternative to the prototypical ones, e.g., poly(vinylidene fluoride) (PVDF),^96^ poly(tetrafluoroethylene),^97^ poly(vinylpyrrolidone),^98^ and poly(vinylidene chloride).^99^ In addition, the ideal binders must be inert toward both the electrode materials and the electrolyte,^16,42,100^ without triggering structural degradations of the device components. Meanwhile, binders must exhibit optimal adhesion properties so that they can be used with a minimal content, while ensuring adequate electrical conductivity of the FSSSC electrodes.^94^

Considering the aforementioned considerations, this work reports the use of 2D metallic niobium disulfide (NbS_2_) nanoflakes, produced by LPE of bulk 2H/3R-NbS_2_ crystals, as functional nanofillers for SPEEK-based composite solid-state electrolytes for FSSSCs. To enhance their functionalities, NbS_2_ nanoflakes were chemically functionalized by linking the thiol group of sodium 3-mercapto-1-propanesulfonate salt (SMPS) molecules to NbS_2_ via S–S bonds or S-vacancy passivation. Thereafter, functionalized NbS_2_ (f-NbS_2_) was incorporated in high-DS SPEEK to form a nanocomposite electrolyte exhibiting high σ (up to 94.4 mS cm^–1^ at room temperature) and optimal mechanical stability (mechanical strength up to 38.3 MPa). These properties resulted in the formation of a robust SO_3_^–^H_3_O^+^ network, leading to an efficient proton transport via the Grotthuss mechanism (dominant channel). The solid-state electrolytes were produced in the form of self-standing membranes, which were directly sandwiched by two electrodes, composed of activated carbon (72 wt %), single/few-layer graphene (8 wt %), carbon black (10 wt %), and SPEEK or PVDF as the binder (10 wt %),^101,102^ to assemble FSSSCs. By doing so, the main FSSSC components, i.e., the electrodes and the solid-state electrolyte, can be fabricated separately, facilitating device manufacturing, but, at the same time, calling for the identification of suitable binders that guarantee the electrical connection between the active materials of the electrodes and the solid-state electrolyte. Either proton-conducting SPEEK or electrically insulating PVDF were used as binders for the electrodes to elucidate the role of ion-conducting binders to fully utilize the active material surface for electrostatic charging/discharging processes. Our results show that optimized FSSSCs combining the f-NbS_2_/SPEEK nanocomposite electrolyte and the proton-conducting SPEEK-based binder can achieve a specific (gravimetric) capacitance (*C*_g_) of 116 F g^–1^ at 0.02 A g^–1^, optimal rate capability (76 F g^–1^ at 10 A g^–1^), and excellent electrochemical stability over galvanostatic charge/discharge (GCD) cycles. Overall, f-NbS_2_ nanoflakes represent promising functional additives for the development of solid-state polymeric electrolytes, which can be directly produced and used in form of a membrane when coupled with ion-conducting binder-based electrodes.

## 2 Results and Discussion

**Figure 1:**
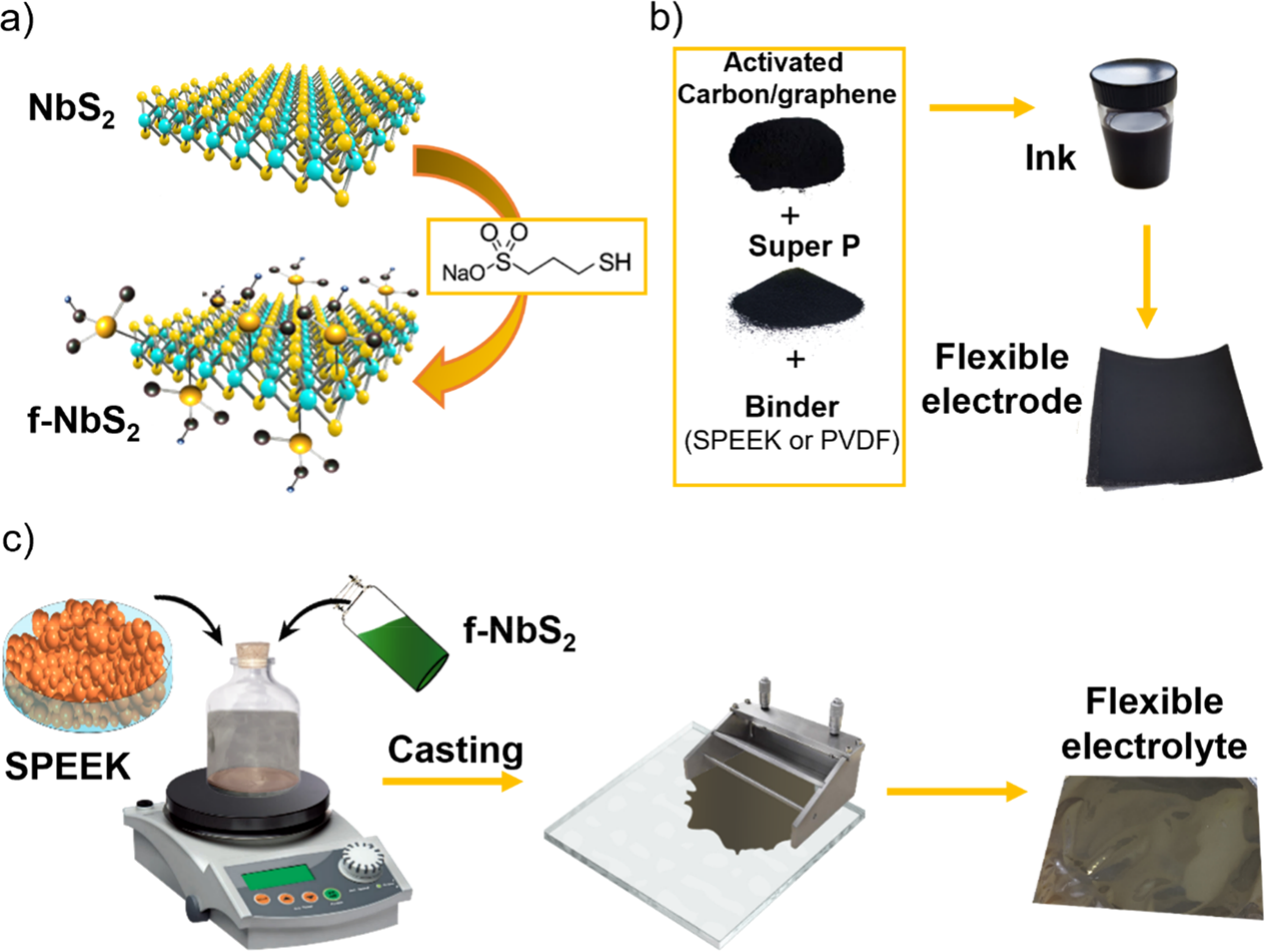
(a) Sketch of the functionalization of NbS_2_ nanoflakes. The thiol group of SMPS molecules was linked to NbS_2_ via S–S bonds or S-vacancy passivation. (b) Sketch of the preparation of the flexible FSSSC electrodes. (c) Sketch of the preparation of the solid-state electrolyte via the incorporation of f-NbS_2_ nanoflakes into the SPEEK matrix.

The NbS_2_ nanoflakes were produced through ultrasonication-assisted LPE and subsequently functionalized using SMPS. The functionalization processes, electrode preparation, and flexible electrolyte preparation are sketched in Figure 1a–c, respectively, and the procedures (including LPE ones) are described in detail in the Supporting Information (SI) Experimental Section. Furthermore, Table S1 lists all the investigated solid-state supercapacitors, which were named *X*:*Y*, in which *X* refers to the binder used for the electrode formulation and *Y* is the (solid-state or liquid) electrolyte. A traditional EDLC using PVDF as the binder and 1 M H_2_SO_4_ as the liquid (aqueous) electrolyte was also assembled and characterized as aqueous EDLC reference.

### 2.1 Morphological and Structural Characterization of Exfoliated NbS_2_ and f-NbS_2_ Nanoflakes

**Figure 2:**
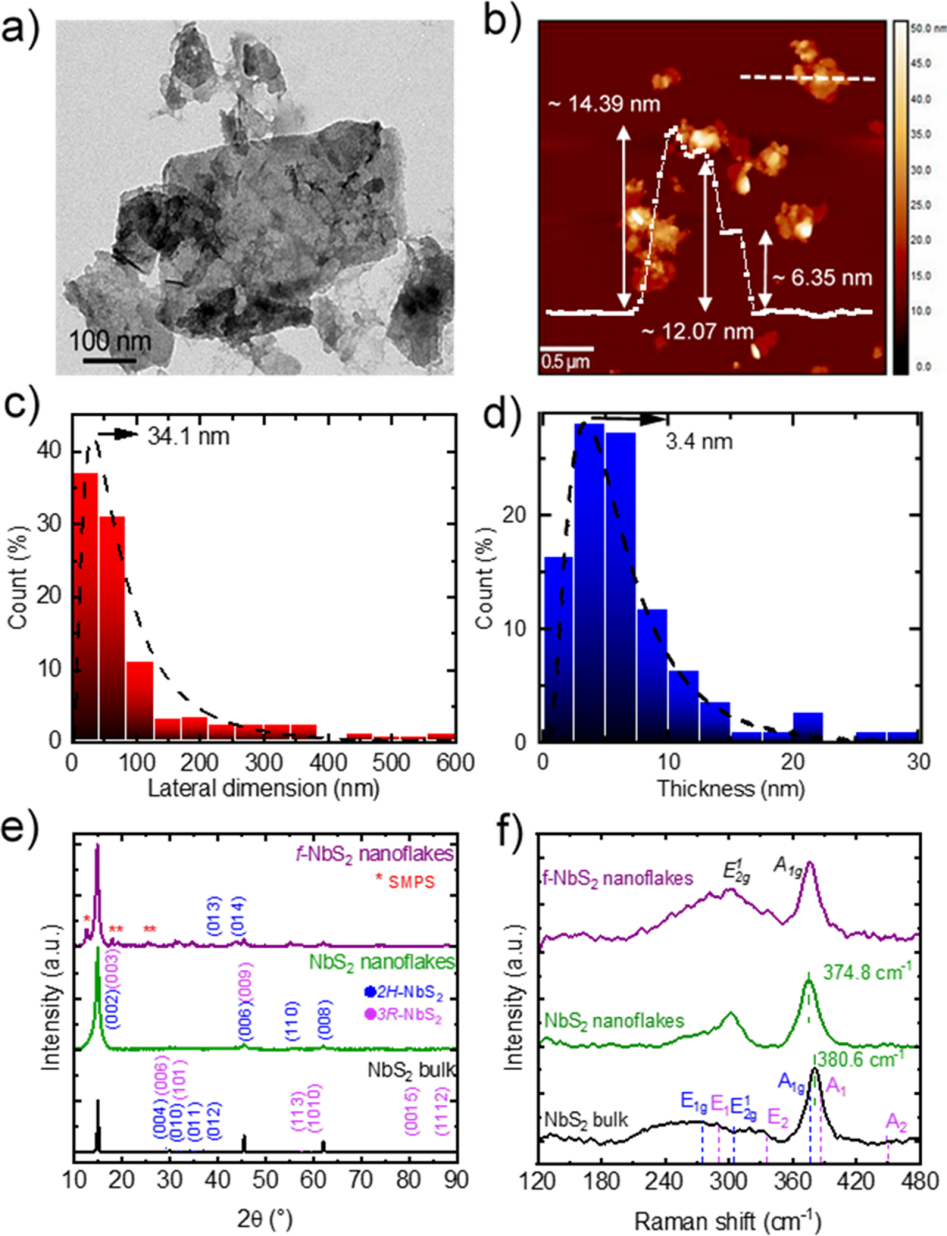
(a) TEM and (b) AFM images of representative f-NbS_2_ nanoflakes. (c) Lateral size and (d) thickness statistical analyses for f-NbS_2_ nanoflakes. (e) XRD patterns and (f) Raman spectra of NbS_2_ bulk crystals, exfoliated NbS_2_, and f-NbS_2_ nanoflakes. The XRD and Raman peaks assigned to the 2H- and 3R-NbS_2_ phases are also shown.

The morphology of the LPE-produced NbS_2_ and f-NbS_2_ nanoflakes was characterized by means of transmission electron microscopy (TEM) and atomic force microscopy (AFM) measurements. Figure 2a shows a bright-field TEM (BF-TEM) image of representative f-NbS_2_ nanoflakes, which display wrinkled surfaces with irregular shapes and sharp edges. Figure 2b reports an AFM image of representative f-NbS_2_ nanoflakes. The height profiles reveal the presence of few-/multilayer flakes, being the measured experimental AFM thickness measured for NbS_2_ monolayers between 0.6 and 0.9 nm, depending on the AFM instrumentation and substrate.^80,103^ According to the TEM data statistical analysis (Figure 2c), the nanoflakes have lateral sizes ranging from 5 to 600 nm, and the lateral size data follows a log-normal distribution peaking at ∼34.1 nm. Meanwhile, the log-normal distribution fitting the AFM thickness data peaks at ∼3.4 nm, revealing the presence of few-layer flakes and monolayers (i.e., thickness <1 nm) (Figure 2d). Notably, the morphology of the functionalized nanoflakes is similar to that observed for the native LPE-produced NbS_2_ flakes, whose characterization is reported in Figure S1.^104^

Figure 2e shows the X-ray diffraction (XRD) patterns of the bulk NbS_2_ crystals, NbS_2_ nanoflakes, and f-NbS_2_ nanoflakes. The as-synthesized bulk NbS_2_ crystals exhibit a structure associated with two different polytypes: the hexagonal 2H phases (space group: *P*6_3_/*mmc*; ICSD card no. 603911), formed by two NbS_2_ layers per unit cell,^105,106^ and hexagonal 3R phases (space group: *R*3*m*; ICSD card no. 51588), which is composed by three NbS_2_ layers.^105,107^ The exfoliated nanoflakes show an intense diffraction peak at around 2θ = 14°, corresponding to the (002) or (003) planes of the 2H and 3R phases, respectively. This peak is broader than the one of the NbS_2_ bulk crystals, due to the reduced crystalline domains of the produced nanoflakes.^72,104^ The absence of characteristic peaks attributed to crystallinity impurities confirms the quality of the exfoliated product, which preserves the crystallinity structure of the basal planes after the LPE process. The diffractogram of the f-NbS_2_ nanoflakes presents characteristic peaks at diffraction angles lower than 30° attributable to the SMPS residuals (space group: *P*1211; ICDS card no. 96-151-4904). Importantly, the positions of the extra XRD peaks observed in the f-NbS_2_ nanoflakes do not match those of the peaks of niobium oxides, suggesting that the f-NbS_2_ nanoflakes preserved the structural properties of the basal planes of the starting nanoflakes.

The structural properties of the produced materials were further evaluated through Raman spectroscopy measurements. According to the group theory for the space group of 2H-NbS_2_^108,109^ and 3R-NbS_2_,^108,110^ the materials display nondegenerate Raman active modes. As shown in Figure 2f, the 2H phase of bulk NbS_2_ shows the E_1g_, E^1^_2g_, and A_1g_ modes at ∼281, ∼303, and ∼380.6 cm^–1^, respectively,^108,109^ while the 3R phase exhibits the E_1_, E_2_, A_1_, and A_2_ modes at ∼296, ∼322, ∼380, and ∼450 cm^–1^, respectively.^108,110,111^ The two peaks at ∼147 and ∼174 cm^–1^ are associated with two-phonon scattering processes in the presence of defects.^103,110,112^ Noteworthy, the peaks related to 2H-NbS_2_ are more pronounced in the exfoliated nanoflakes than the bulk crystals, suggesting that the exfoliation process promotes a 3R- to 2H- phase conversion, in agreement with previous literature.^103,104^ Moreover, the A_2_ mode of the 3R-NbS_2_ is red-shifted from ∼450 cm^–1^ in the NbS_2_ crystal to ∼435 cm^–1^ in the nanoflakes, because the interlayer van der Waals forces relax with decreasing the number of layers.^104,113^ In addition, the Raman analysis indicates that the exfoliation and functionalization of NbS_2_ nanoflakes does not significantly change the crystalline structure of the basal planes of bulk crystals.

### 2.2 Morphological Characterization of Electrolyte

**Figure 3:**
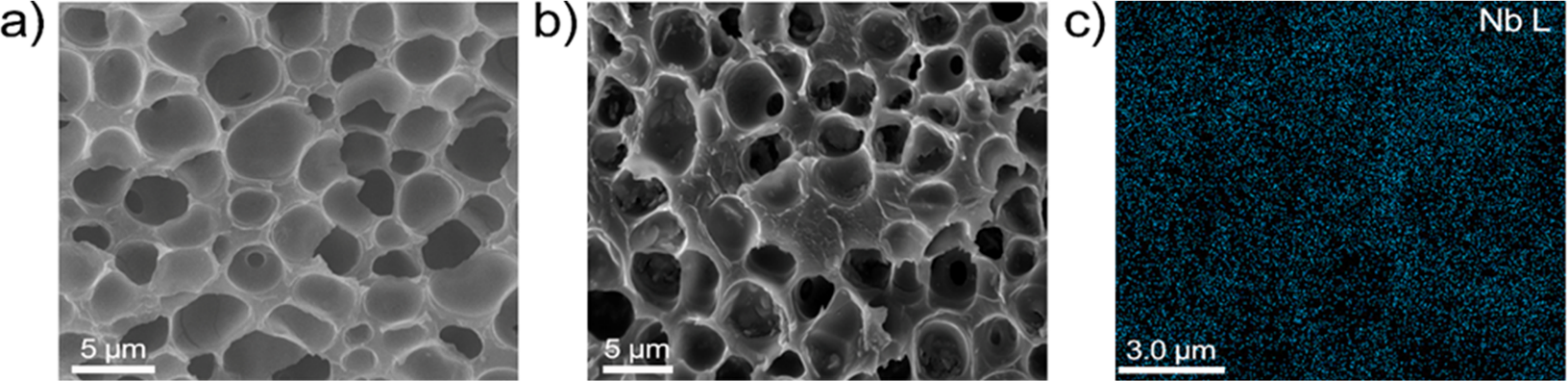
Cross-sectional SEM images of (a) SPEEK and (b) 2.5%-f-NbS_2_:SPEEK, respectively. (c) EDX map of Nb (M line at 2.18 keV) for 2.5%-f-NbS_2_:SPEEK.

The morphology and chemical characteristics of the as-produced composite solid-state electrolytes (hereafter named *x*%-f-NbS_2_:SPEEK, in which *x*% indicates the wt % of f-NbS_2_ nanoflakes in the electrolyte) were evaluated through energy-dispersive X-ray spectroscopy (EDX)-coupled scanning electron microscope (SEM) measurements. Figure 3a,b depicts the cross-sectional SEM images of the pristine SPEEK and 2.5%-f-NbS_2_:SPEEK electrolytes, respectively. The SPEEK electrolyte exhibits a porous morphology, made of pores with lateral dimensions between 1 and 6 μm. This peculiar morphology is associated with the sulfonation process that introduces hydrophilic −SO_3_H groups causing the reorganization of the hydrophobic backbone formed by SPEEK chains (Figure 3a). The nanocomposite electrolyte is also porous, and its pores have a coarse surface (Figure 3b). The pore coarseness may originate from the spatial confinements of the polymeric materials forced by f-NbS_2_ nanoflakes.^114,115^ Thanks to the capability of sulfonated groups to form electrostatic interactions with sulfonated polymers,^115−117^ the f-NbS_2_ nanoflakes are homogeneously distributed within the SPEEK matrix. This uniformity is due to the hydrogen bonds between sulfonated groups of the SPEEK and f-NbS_2_ nanoflakes, resulting in ion-conducting pathways that facilitate ion transportation through the nanocomposite electrolyte compared to the pristine SPEEK.^118^Figure 3c reports the EDX map measured for Nb, enabling the further evaluation of the f-NbS_2_ nanoflakes dispersion in the SPEEK nanocomposite electrolyte. The data indicate that the nanoflakes are uniformly distributed within the polymeric matrix. The SPEEK surrounding the nanoflakes ensures that the latter are electrically isolated, excluding their electrical contact with the active material of the electrodes in supercapacitor devices (as shown hereafter). Furthermore, the uniform distribution of the f-NbS_2_ nanoflakes and their chemical interactions with the polymeric matrix reinforce the mechanical and thermal properties of the prepared solid-state electrolytes.^119^

**Figure 4:**
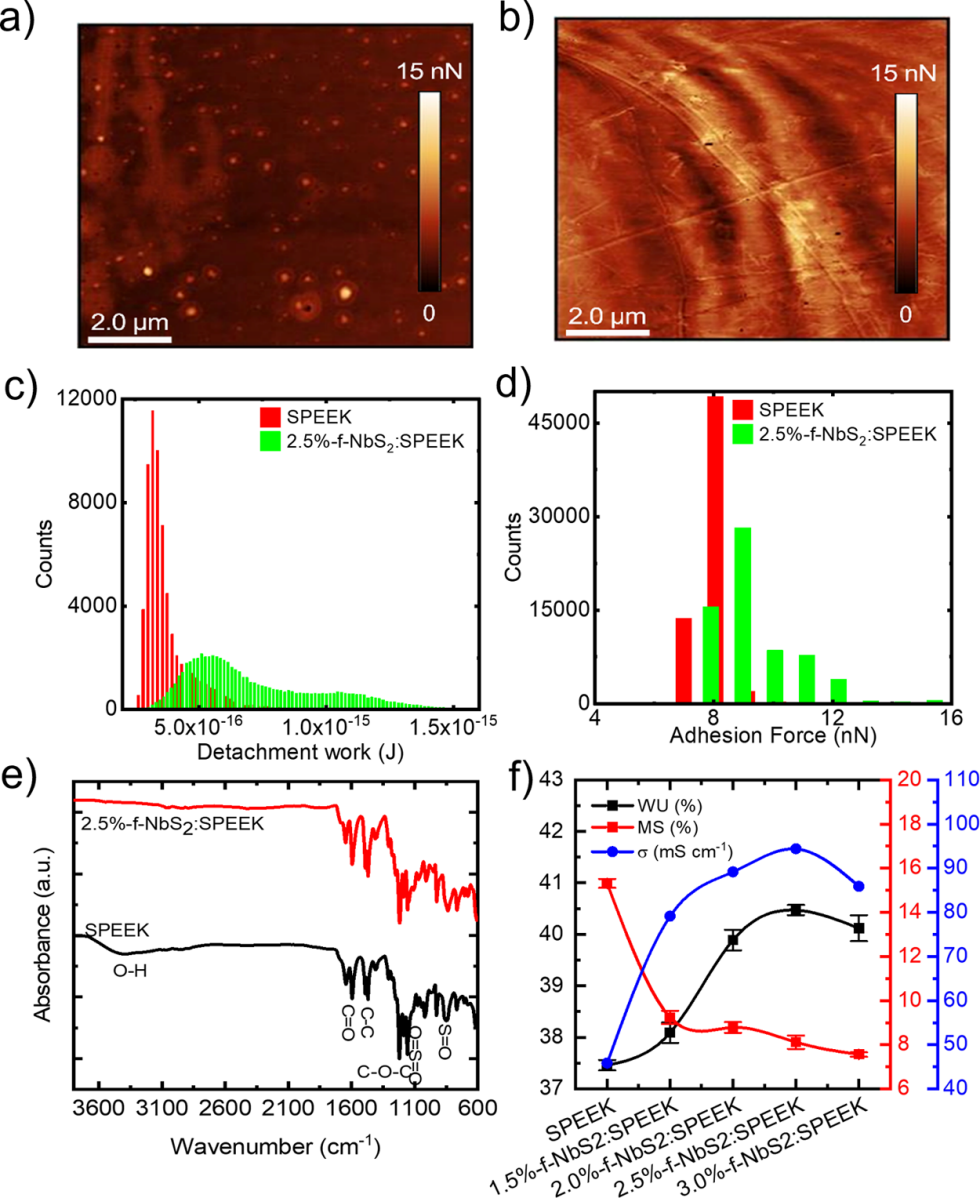
Adhesion force maps measured by AFM for (a) SPEEK and (b) 2.5%-f-NbS_2_:SPEEK in humid ambient air, respectively, and the corresponding (c) detachment work and (d) adhesion force distributions. (e) FTIR spectra of SPEEK and 2.5%-f-NbS_2_:SPEEK electrolytes. (f) WU, MS, and σ of the prepared solid-state electrolytes.

The hydrophilic–hydrophilic nanophase separation in proton-conducting polymers strongly influences several material properties, including WU and σ. Moreover, according to the Lennard–Jones force–separation relation,^120−122^ the AFM measurements can be used to identify hydrophilicity/hydrophobicity domains in the solid-state electrolytes.^123^ In fact, the adhesion force between the AFM tip and the membrane surface is dictated by the capillary force, which depends on the hydrophilic/hydrophobic properties of the investigated materials.^124,125^ In addition, the chemical specificity (e.g., the presence of functional groups) of the solid-state electrolytes can also affect the pull-off force at the nano/microscale,^120,121^ providing a quantitative evidence of hydrophilic polar chemical species. Figure 4a,b shows the adhesion force maps measured for SPEEK and 2.5%-f-NbS_2_:SPEEK electrolytes, respectively. In addition, Figure 4c,d shows the corresponding detachment work (i.e., the work needed to detach the AFM tip from the sample) and adhesion force distributions, respectively. The mean detachment works are (0.33 ± 0.23) × 10^–15^ and (0.52 ± 0.13) × 10^–15^ J for SPEEK and 2.5%-f-NbS_2_:SPEEK, respectively, which correspond to mean adhesion forces of 8.12 ± 0.32 and 8.93 ± 0.21 nN, respectively. These data indicate that hydrophilic domains and functional polar groups in 2.5% f-NbS_2_:SPEEK electrolyte are more abundant compared to SPEEK, positively affecting electrolyte WU and σ. Fourier-transform infrared (FTIR) spectroscopy measurements were carried out to further evaluate the chemical specificity of the investigated solid-state electrolytes. Figure 4e reports the FTIR spectra of the SPEEK and the 2.5%-f-NbS_2_:SPEEK electrolytes in the 600–3800 cm^–1^ range. The absorption bands at 3422 cm^–1^ in the SPEEK electrolyte and 3060 cm^–1^ in the 2.5%-f-NbS_2_:SPEEK nanocomposite electrolyte are ascribed to hydroxyl groups.^126^ The symmetric absorption peak of the C=O groups appears in the SPEEK spectrum at 1644 cm^–1^, in agreement with the literature.^127^ In addition, SPEEK shows characteristic absorption bands at ∼1220 and ∼1490 cm^–1^. These bands are ascribed to the symmetric stretching of the C–O–C and C–C benzene rings, respectively.^128^ The peaks observed at 1020, 1076, and 1250 cm^–1^ are assigned to the asymmetric and symmetric stretching vibrations of O=S=O and the stretching vibration of S=O in −SO_3_H groups, respectively, confirming the PEEK sulfonation.^129^ In 2.5%-f-NbS_2_:SPEEK, the presence of the f-NbS_2_ is assessed by the analysis of the intensity and shape of the bands related to asymmetric and symmetric O=S=O bonds. In particular, the changes observed in the FTIR spectrum are ascribed to the more abundant −SO_3_H groups in the nanocomposite structure provided by f-NbS_2_ nanoflakes, which can have a direct condensation reaction with the sulfonic acid group of the SPEEK.^126^ Furthermore, the attenuation of −OH peak around 3350 cm^–1^ in the nanocomposite electrolyte, compared to the pristine SPEEK electrolyte, may be associated with the strong hydrogen bonds between the −SO_3_H groups of the SPEEK and the f-NbS_2_ nanoflakes.^126^

To assess the physicochemical properties of the solid-state electrolytes, the WU and MS parameters were measured, verifying their influence on the electrolyte ionic conductivity and stability.^75,130^Figure 4f reports the WU and MS of the produced solid-state electrolytes. In particular, the WU of the nanocomposite electrolyte is higher than the one of the pristine SPEEK and increases significantly with increasing the f-NbS_2_ nanoflakes content from 37.5% for pristine SPEEK to 40.5% for 2.5%-f-NbS_2_:SPEEK. This effect is ascribed to the superior hydrophilicity of the nanocomposite electrolyte due to the additional hydrophilic −SO_3_H groups of the f-NbS_2_ nanoflakes. Therefore, the formation of hydrogen bonds between −SO_3_H groups of the f-NbS_2_ nanoflakes and the water molecules increases the WU of the nanocomposite electrolytes compared to pristine SPEEK.^131^ However, by increasing the content of f-NbS_2_ nanoflakes to more than 2.5 wt %, the WU of the nanocomposite electrolyte decreases, as a consequence of the nanoflakes aggregation. Contrary to the WU, the MS of the solid-state electrolytes decreases with increasing the f-NbS_2_ content, indicating that f-NbS_2_ nanoflakes improve the dimensional stability of the solid-state electrolytes.^132^ Generally, despite the presence of large amounts of hydrophilic −SO_3_H groups that absorb water in its structure, SPEEK intrinsically shows a limited MS with increasing the content of free water molecules. According to this behavior, the produced electrolytes exhibit MS values ranging from 15.3% for SPEEK to 7.6% for 3.0%-f-NbS_2_:SPEEK, at room temperature. Furthermore, the network of hydrogen bonds originated by chemical interaction between f-NbS_2_ nanoflakes and SPEEK chains can limit the mobility of the SPEEK chains in the nanocomposite electrolytes.^66^ The superior dimensional stability of the nanocomposite electrolytes compared to the pristine one is attractive for stable supercapacitor operation over time and prospectively can be a key-property to maximize the volumetric performance of FSSSCs.^133^ The electrochemical performances of the solid-state electrolytes are strongly determined by their σ,^134^ which was measured at room temperature (Figure 4f). The composite solid-state electrolytes exhibit higher σ than SPEEK. In particular, 2.5%-f-NbS_2_:SPEEK achieved the maximum σ of 0.094 S cm^–1^, which is ∼2 times higher than the value obtained for SPEEK (0.046 S cm^–1^). Generally, the σ of the proton-conducting polymers depends on the number of ion-conducting groups.^131^ The chemical interaction between −SO_3_H groups of the f-NbS_2_ nanoflakes and SPEEK provides a proton-transferring SO_3_^–^H_3_O^+^ network, in which protons can be transported through the electrolyte via the Grotthuss mechanism.^135^ Similar to the WU, also the σ of the produced electrolytes increases with increasing the f-NbS_2_ content until 2.5 wt %. In fact, the formation of nanoflake aggregates for excessive NbS_2_ content (above 2.5 wt %) deteriorates the proton-transferring SO_3_^–^H_3_O^+^ network, cutting proton-transporting pathways.^136−138^Figure S2 reports the properties measured on composite solid-state electrolytes produced by replacing f-NbS_2_ nanoflakes with functionalized 2H-MoS_2_ (f-MoS_2_) nanoflakes. Importantly, (2D) 2H-MoS_2_ is one of the most investigated (2D) group-6 TMDs, displaying semiconductive properties that are substantially different from the metallic ones expressed by NbS_2_ nanoflakes. Noteworthy, the metallicity of the TMDs plays a primary role in the electron transfer between TMDs and reactant precursors used for the functionalization of TMDs.^66^ Indeed, previous studies showed that the metallic properties of TMDs can facilitate their functionalization,^87^ thus, in our case, increasing the amount of the −SO_3_ groups. According to the above consideration, the Figure S2 data show that the f-MoS_2_-based solid-state electrolytes reached a lower maximum σ (74.8 mS cm^–1^) compared to those based on f-NbS_2_ nanoflakes (94.4 mS cm^–1^). This suggests that a less robust SO_3_^–^H_3_O^+^ network (leading to a less efficient Grotthuss mechanism) is established for the f-MoS_2_-based nanocomposites compared to those of f-NbS_2_-based ones, which is consistent with the less effective functionalization of the semiconducting 2H-MoS_2_ nanoflakes compared to metallic NbS_2_ nanoflakes.

For the realization of practical FSSSCs, the mechanical properties of solid-state electrolytes, including elongation at break value and Young’s modulus, play a key role. Figure S3a shows the stress–strain curves measured for SPEEK and 2.5%-f-NbS_2_:SPEEK electrolytes. The pristine SPEEK exhibits a tensile stress of 26.7 MPa and an elongation at break value of 7.27% at room temperature. Instead, 2.5%-f-NbS_2_:SPEEK electrolyte achieves tensile stress and elongation at break values of 30.3 MPa and 5.2%, respectively, at room temperature. The extrapolated Young’s moduli are 826.5 and 1066.3 MPa for SPEEK and 2.5%-f-NbS_2_:SPEEK, respectively. Overall, these data (summarized in Table S2) support that the nanocomposite electrolytes have a mechanical strength higher than SPEEK because of the strong interaction between the functional groups of polymeric chains and nanoflakes, as well as the mechanical properties of the latter.^139,140^ In addition, beyond the mechanical features, the thermal properties and residual water can influence the reliability and the electrochemical performances of a solid-state electrolyte.^141^ The thermal behavior of the SPEEK and 2.5%-f-NbS_2_-SPEEK electrolytes was investigated via TGA analysis in N_2_ atmosphere. As reported in Figure S3b, three main steps can be observed in the TGA curves: the first one, between ∼30–105 °C, can be attributed to the excretion of the residual water molecules poorly bonded to the SPEEK (or f-NbS_2_ nanoflakes), i.e., free water.^142^ Importantly, the TGA data indicate that the content of free water in 2.5%-f-NbS_2_-SPEEK (5.8%) is inferior to that of SPEEK (9.8%). This means that, in 2.5%-f-NbS_2_-SPEEK, water molecules are mainly bonded to the composite polymer through hydrogen bonds, which is consistent with our previous physicochemical characterization (Figure 4f). Importantly, these data also confirm that the superior σ of the composite solid-state electrolytes compared to SPEEK is not related to the presence of residual free water molecules (associated with vehicle mechanism), but to the ability of the SO_3_^–^H_3_O^+^ network to transfer protons via Grotthuss mechanism. Between ∼120–280 °C, the weight loss is mainly associated with the decomposition of the sulfonic groups (−SO_3_^–^). In addition, beyond 150 °C, thermal energy can break the hydrogen bonds between (bonded) water molecules and SPEEK or f-NbS_2_ nanoflakes, enabling the evaporation of remaining water molecules. Since the WUs of the composite solid-state electrolytes are higher than that of SPEEK (Figure 4f), in the 120–210 °C temperature range, the weight loss of 2.5%-f-NbS_2_-SPEEK is higher than the one of pristine SPEEK. Lastly, the weight loss starting at temperatures higher than 450 °C is ascribed to the degradation of the polymer chains.^131^ At temperatures higher than 600 °C, the higher weight retention measured for the 2.5%-f-NbS_2_-SPEEK compared to that of the pristine SPEEK is likely associated with the presence of f-NbS_2_ nanoflakes or related nonvolatile decomposition products.^143^

### 2.3 Electrochemical Characterization

**Scheme 1:**
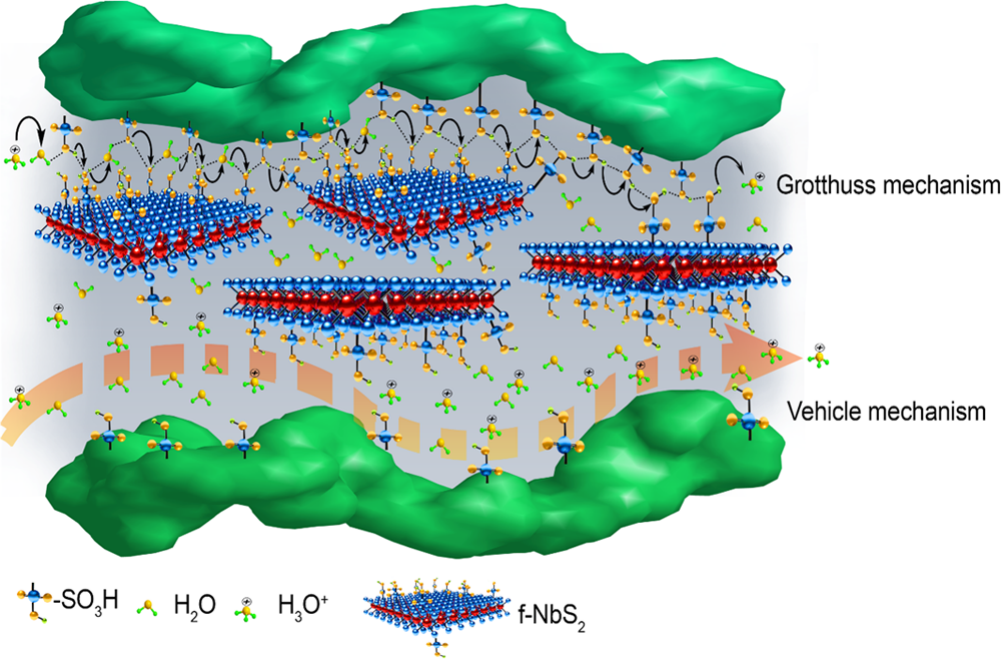
Ion Transport Mechanisms through the Composite Solid-State Electrolyte: Grotthuss (Primary) Mechanism and Vehicle (Secondary) Mechanisms

**Figure 5:**
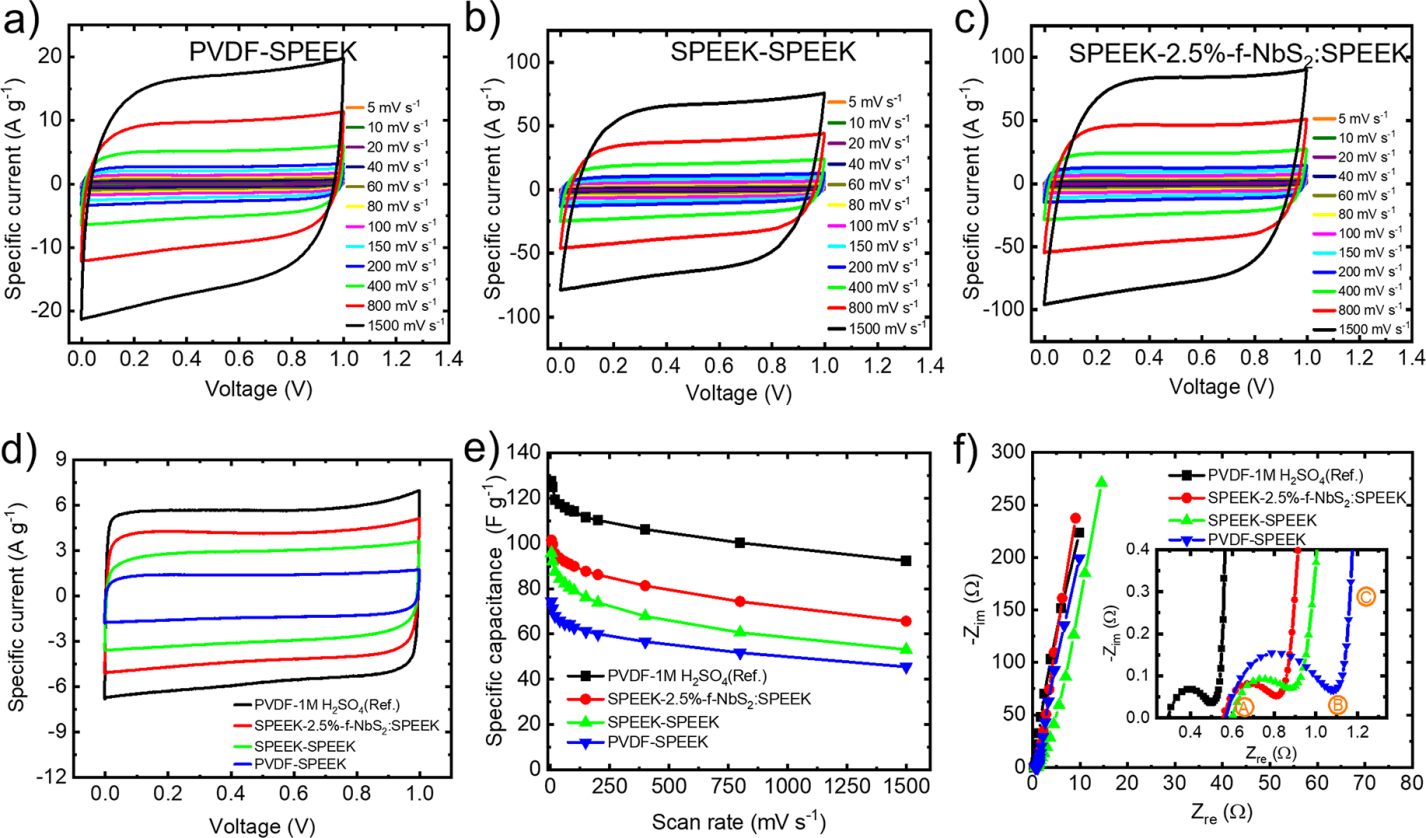
Electrochemical characterization of the investigated solid-state supercapacitors and 1 M H_2_SO_4_-based EDLC reference. CV curves measured for the (a) PVDF-SPEEK, (b) SPEEK-SPEEK, and (c) SPEEK-2.5%-f-NbS_2_:SPEEK, acquired at voltage scan rates ranging from 5 to 1500 mV s^–1^. (d) CV curves of the investigated EDLCs acquired at 100 mV s^–1^ voltage scan rate. (e) Electrode *C*_g_ of the investigated EDLCs as a function of the voltage scan rate, extrapolated from the CV analysis. (f) Nyquist plots of the investigated EDLCs. The inset panel shows the enlargement of the high-frequency regions of the Nyquist plots.

The electrochemical performances of our solid-state electrolytes for supercapacitors were evaluated in symmetric configurations using carbonaceous electrodes. The electrodes, composed by activated carbon (72 wt %), single/few-layer graphene (8 wt %), carbon black (10 wt %), and polymeric binder (10 wt %), were fabricated following the protocols reported in our previous works (see SI, Experimental Section).^101,102^ Beyond the choice of the solid-state electrolyte, the identification of suitable binders is also needed to ensure a continuous electrical pathway from the solid-state electrolyte and the surface of the electrode active materials,^90−93^ leading to a high-capacitance electrical double layer.^77,78^ Thus, both proton-conducting SPEEK and electrically insulating PVDF were evaluated as binders for the electrodes of our solid-state supercapacitors. Figure S4a,b shows the surface and cross-sectional SEM images of a representative electrode prepared with SPEEK binder and using carbon cloth as the current collector. The carbon cloth is made of interconnected carbon fibers that are coated by the electrode materials (i.e., activated carbon, single/few-layer graphene, and binder). Figure 5a–c shows the CV curves measured for the PVDF-SPEEK, SPEEK-SPEEK, and SPEEK-2.5%-f-NbS_2_:SPEEK devices, respectively, at voltage scan rates ranging from 5 to 1500 mV s^–1^ in the voltage window of 0–1 V. The CV curve measured for the reference device, i.e., PVDF-1 M H_2_SO_4_, is reported in Figure S5a. All the CV curves maintained nearly rectangular shapes with increasing the voltage scan rate from 5 up to 1500 mV s^–1^, indicating satisfactory rate capabilities.^144^ The absence of peaks ascribable to (Faradaic) redox reactions confirmed the capacitive behavior expected for EDCLs.^145,146^ To evaluate the synergistic effect of the binders and electrolytes on the electrochemical performance of FSSSCs, Figure 5d shows the CV curves measured for PVDF-SPEEK, SPEEK-SPEEK, and SPEEK-2.5%-f-NbS_2_:SPEEK at 100 mVs^–1^. The use of SPEEK as binder increases the specific current compared to those recorded for the device using PVDF as binder (i.e., PVDF-SPEEK). In the latter, although the solid-state electrolyte or electrodes were not prewetted by adding small amounts of liquid electrolyte, residual free water molecules in the solid-state electrolyte (see TGA analysis, Figure S3b) can move from the electrolyte to the electrode, allowing the devices to function even when using an electrically insulating PVDF binder (even though with lower performance compared to the SPEEK binder-based devices). Figure 5e reports the electrode *C*_g_ for the devices, calculated from the CV analysis at different voltage scan rates. The device with SPEEK-2.5%-f-NbS_2_:SPEEK nanocomposite electrolyte exhibits the highest *C*_g_ (101 F g^–1^ at 5 mV s^–1^). The optimal rate capability of this device can be associated with the high σ (94 mS cm^–1^) of the solid-state electrolyte in the presence of f-NbS_2_ nanoflakes (promoting an efficient Grotthuss mechanism), as well as to the efficient transport of ions toward the electrolyte-active material interface in the presence of SPEEK binder.^147^ The latter permits the ions in the nanocomposite electrolyte to diffuse toward the proximity of the active material surface, maximizing the double layer capacitance.^148,149^ Noteworthy, free water molecule content in composite solid-state electrolytes is less than that of pristine SPEEK (see TGA analysis, Figure S3b), excluding that the vehicle mechanism is responsible of the superior σ of our SPEEK-2.5%-f-NbS_2_:SPEEK electrolyte, which is instead mainly determined by the Grotthuss (primary) mechanism (see Scheme 1).

Electrochemical impedance spectroscopy (EIS) measurements were carried out to evaluate the resistance contribution of the electrolytes, as well as the charge transfer resistances (*R*_ct_) at the binder–electrolyte interfaces.^50^ Typically, the Nyquist plot (i.e., −Im[*Z*] vs Re[*Z*], in which *Z* is the complex impedance) of a liquid electrolyte-based EDLC consists of a semicircle at high frequencies between points A and B, a nonvertical quasi-straight line at intermediate frequencies between points B and C, and a nearly vertical line at low frequencies beyond point C (as also shown in our EIS data, Figure 5f). However, these contributions can overlap, complicating the extrapolation of reliable parameters. Previous studies demonstrated that the diameter of the semicircles between A and B (*R*_AB_) is associated with interfacial resistance of the current collector/electrode interface, while the intersection of the Nyquist plot and the *x*-axis (i.e., *Z*_re_-axis) at the highest frequency (*R*_A_) is associated with the ionic resistance of the electrolyte and the electronic resistance of the electrodes.^150^ In solid-state supercapacitors, the *R*_ct_ at the binder-electrolyte interfaces can also contribute to the *R*_AB_.^151^ As listed in Table 1, the *R*_A_ extrapolated for SPEEK-2.5%-f-NbS_2_:SPEEK is 0.55 Ω, which is lower than those measured for PVDF-SPEEK and SPEEK-SPEEK, and only 1.9 times higher than the *R*_A_ measured for an aqueous 1 M H_2_SO_4_-based EDLCs. This trend may be ascribed to the higher σ of the nanocomposite electrolyte compared to SPEEK. The *R*_AB_ of PVDF-SPEEK (0.54 Ω) is clearly higher than those extrapolated for SPEEK-SPEEK and SPEEK-2.5%-f-NbS_2_:SPEEK (0.31 Ω and 0.28 Ω, respectively), indicating that the use of an electrically insulting binder can negatively affect the ion transport from the solid-state electrolyte toward the active material surface, leading to an increase of *R*_ct_.^152^ Contrary, the ion-conducting characteristics of the SPEEK binder can improve the ion transport from the electrolyte toward the electrode active materials, enabling a high-capacitance double layer formation.^37,153^ Lastly, the low-frequency regions of Nyquist plots of the devices exhibit nearly vertical straight lines (i.e., parallel to the *y*-axis, i.e., the −*Z*_im_-axis). These lines confirm the capacitive behavior of the EDCLs, and can be represented by the relation *Z*_im_ = −1/(2π*f* × *C*), in which *C* is the device capacitance and *f* is the frequency.^4,154^ The intersection of this straight line with the *Z*_re_-axis is typically associated with the overall equivalent series resistance (ESR) of the device, which can be also extrapolated by the voltage drop observed in the first stage of the galvanostatic charging and discharging of the device (as shown hereafter).

**Table 1:** Comparison between the Resistance Metrics, i.e., *R*_A_ and *R*_AB_, of the Investigated Supercapacitors, Extrapolated by the Analysis of Their Nyquist Plots

samples	*R*_A_ (Ω)	*R*_AB_ (Ω)
PVDF-1 M H_2_SO_4_ (ref.)	0.29	0.24
PVDF-SPEEK	0.57	0.54
SPEEK-SPEEK	0.59	0.306
SPEEK-2.5%-f-NbS_2_:SPEEK	0.56	0.28

**Figure 6:**
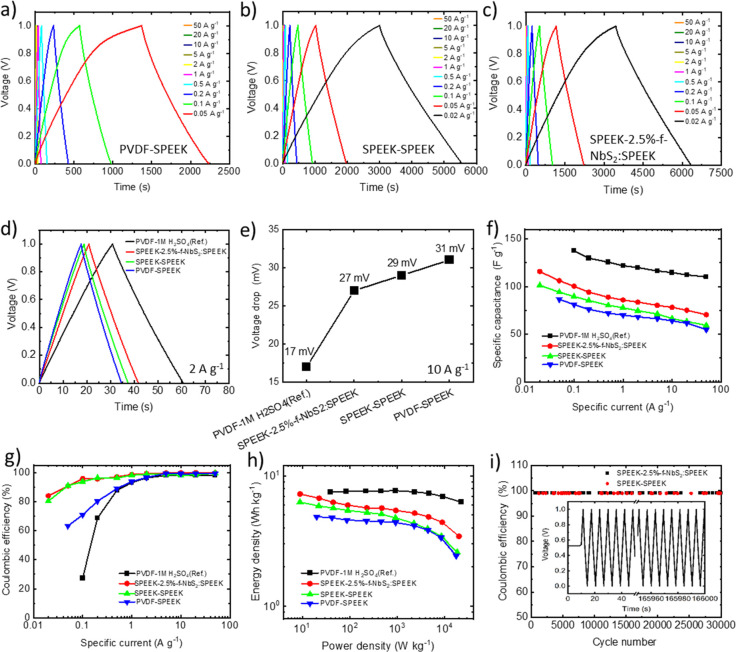
Electrochemical characterization of the prepared solid-state supercapacitors. GCD curves acquired at specific currents ranging from 0.02 to 50 A g^–1^ measured for (a) PVDF-SPEEK, (b) SPEEK-SPEEK, and (c) SPEEK-2.5%-f-NbS_2_:SPEEK. (d) GCD curves measured for the device at 2 A g^–1^. (e) *V*_drop_ measured from GCD curves at specific current of 10 A g^–1^. (f) Electrode specific *C*_g_ and (g) Coulombic efficiency vs specific current plots and (h) Ragone plots measured for the investigated solid-state supercapacitor electrodes. (i) Stability of SPEEK-2.5%-f-NbS_2_:SPEEK and SPEEK-SPEEK over 30 000 charge/discharge cycles (inset: charge/discharge cycles at 10 A g^–1^).

The performances of the investigated solid-state supercapacitors were further evaluated by performing GCD measurements at various specific currents, ranging from 0.02 to 50 A g^–1^. The GCD curve measured for the reference device, i.e., PVDF-1 M H_2_SO_4_ is reported in Figure S5b. Figure 6a–c shows the GCD curves measured for PVDF-SPEEK, SPEEK-SPEEK, and SPEEK-2.5%-f-NbS_2_:SPEEK samples, respectively. The GCD curves exhibit nearly triangular shapes for all the investigated specific currents, confirming the capacitive behavior of the devices.^37^ The voltage drop during the initial stage of charge and discharge (here referred as *V*_drop_) is associated with the resistive losses caused by device ESR, being proportional to the applied currents, i.e., *V*_drop_ = *I* × ESR.^102,155,156^Figure 6d reports the comparison between the GCD curves obtained for different device configurations at the specific current of 2 A g^–1^. The calculated *V*_drop_ are 17, 27, 29, and 31 mV for PVDF-1 M H_2_SO_4_, PVDF-SPEEK, SPEEK-SPEEK, and SPEEK-2.5%-f-NbS_2_:SPEEK cells, respectively (Figure 6e). These data indicate that the combination of high-σ 2.5%-f-NbS_2_:SPEEK electrolyte and SPEEK binder can limit the *V*_drop_ of solid-state supercapacitors, improving their rate capability performance.^144^ The rate capability of the investigated devices was further evaluated by analyzing their electrode *C*_g_ as a function of the specific current.^157^ As shown in Figure 6f, the electrode *C*_g_ decreases with increasing the specific current for all devices, even if the combination of nanocomposite solid-state electrolyte and SPEEK binder improves the device rate capability of solid-state supercapacitors, confirming the beneficial roles of f-NbS_2_ nanoflakes and ion-conducting binder (in accordance with the CV analysis). Among our devices, SPEEK-2.5%-f-NbS_2_:SPEEK achieved the maximum *C*_g_ of 116 F g^–1^ at 0.02 A g^–1^, which is almost equal to the value calculated from the CV curve at 5 mV s^–1^ and approaches that recorded for PVDF-1 M H_2_SO_4_ reference (137 F g^–1^ at 0.1 A g^–1^). Importantly, our solid-state devices were able to efficiently operate at specific currents as low as 0.05 and 0.02 A g^–1^, at which they showed the highest *C*_g_. Contrary, at such specific currents, current leakage and parasitic reactions result in a poor capacitive behavior of the PVDF-1 M H_2_SO_4_ device, impeding its operation in the investigated voltage window. At 50 A g^–1^, SPEEK-2.5%-f-NbS_2_:SPEEK retained 66.5% of the *C*_g_ measured at 0.05 A g^–1^. The *C*_g_ retention passing from 0.05 to 50 A g^–1^ was higher than that of PVDF-SPEEK (63.3%), which is consistent with the superior σ of the nanocomposite electrolyte compared to SPEEK, as well as the ability of the SPEEK binder to transport ions from the electrolyte toward the surface of active materials. According to Figure 6g, the Coulombic efficiency of the f-NbS_2-_free devices drops to values lower than 80.35% at the specific current of 0.02 A g^–1^, confirming the presence of current leakage at the interface between the electrode and the electrolyte.^158,159^ Nevertheless, the presence of 2.5%-f-NbS_2_:SPEEK reduces the current leakage, enabling the device to operate at 0.02 A g^–1^ with a Coulombic efficiency of 83.9%. At specific currents higher than 0.5 A g^–1^, the Coulombic efficiencies remain higher than 97% for all the investigated devices. Figure 6h reports the energy density vs power density plots (i.e., Ragone plots) measured for the investigated supercapacitors. As expected, compared to SPEEK-SPEEK and PVDF-SPEEK, the SPEEK-2.5%-f-NbS_2_:SPEEK configuration shows superior energy/power density characteristics. In particular, its energy density is as high as 6.7 Wh kg^–1^ at 22.8 kW kg^–1^, which is 15% higher than the one of SPEEK-SPEEK (5.8 Wh kg^–1^) and 38% higher than the one of PVDF-SPEEK (4.85 Wh kg^–1^). Also, SPEEK-2.5%-f-NbS_2_:SPEEK reached a maximum energy density of 7.2 Wh kg^–1^ at 9.0 kW kg^–1^ power density, while the energy density recorded at the highest power density of 20.1 kW kg^–1^ is 3.4 Wh kg^–1^. Overall, these data further indicate that the incorporation of f-NbS_2_ nanoflakes into SPEEK-based solid-state electrolytes can improve the performances of the resulting solid-state supercapacitors.^145^ Moreover, the performances (i.e., *C*_g_, energy density and power density) achieved for our optimized devices are competitive with those reported in the relevant literature for solid-state supercapacitors (see Table S3).

**Figure 7:**
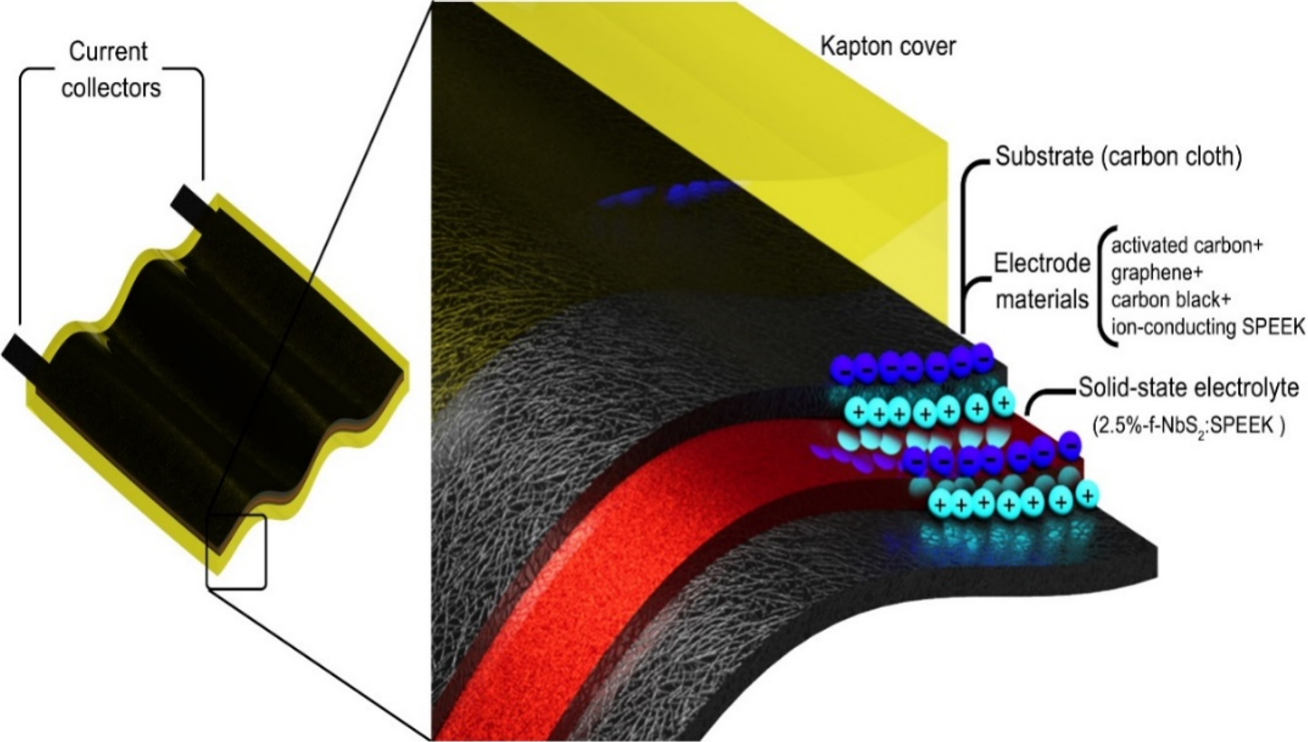
Schematic illustration of the prepared FSSSCs, based on SPEEK-2.5%-f-NbS_2_:SPEEK composite electrolyte, proton-conducting SPEEK as electrode binder, and flexible current collectors (carbon cloths). The FSSSCs were protected by a Kapton-based packaging.

Long cycle life is one of the practical requirements for solid-state supercapacitors.^153,160,161^ To evaluate their cycling stability, SPEEK-SPEEK and SPEEK-2.5%-f-NbS_2_:SPEEK were cycled at a constant specific current of 10 A g^–1^ for 30 000 cycles. As shown in Figure 6i, the SPEEK-SPEEK and SPEEK-2.5%-f-NbS_2_:SPEEK samples showed excellent cycle stability, retaining more than 99.1% of the initial *C*_g_ after 30 000 GCD cycles. Furthermore, almost linear charge and discharge profiles were observed at 10 A g^–1^ for SPEEK-2.5%-f-NbS_2_:SPEEK (inset Figure 6i), confirming their nearly ideal EDLC behavior during the time. Based on the previous electrochemical characterizations, our best performance solid-state electrolyte-binder combination was used to fabricate FSSSCs, as schematically illustrated in Figure 7.

**Figure 8:**
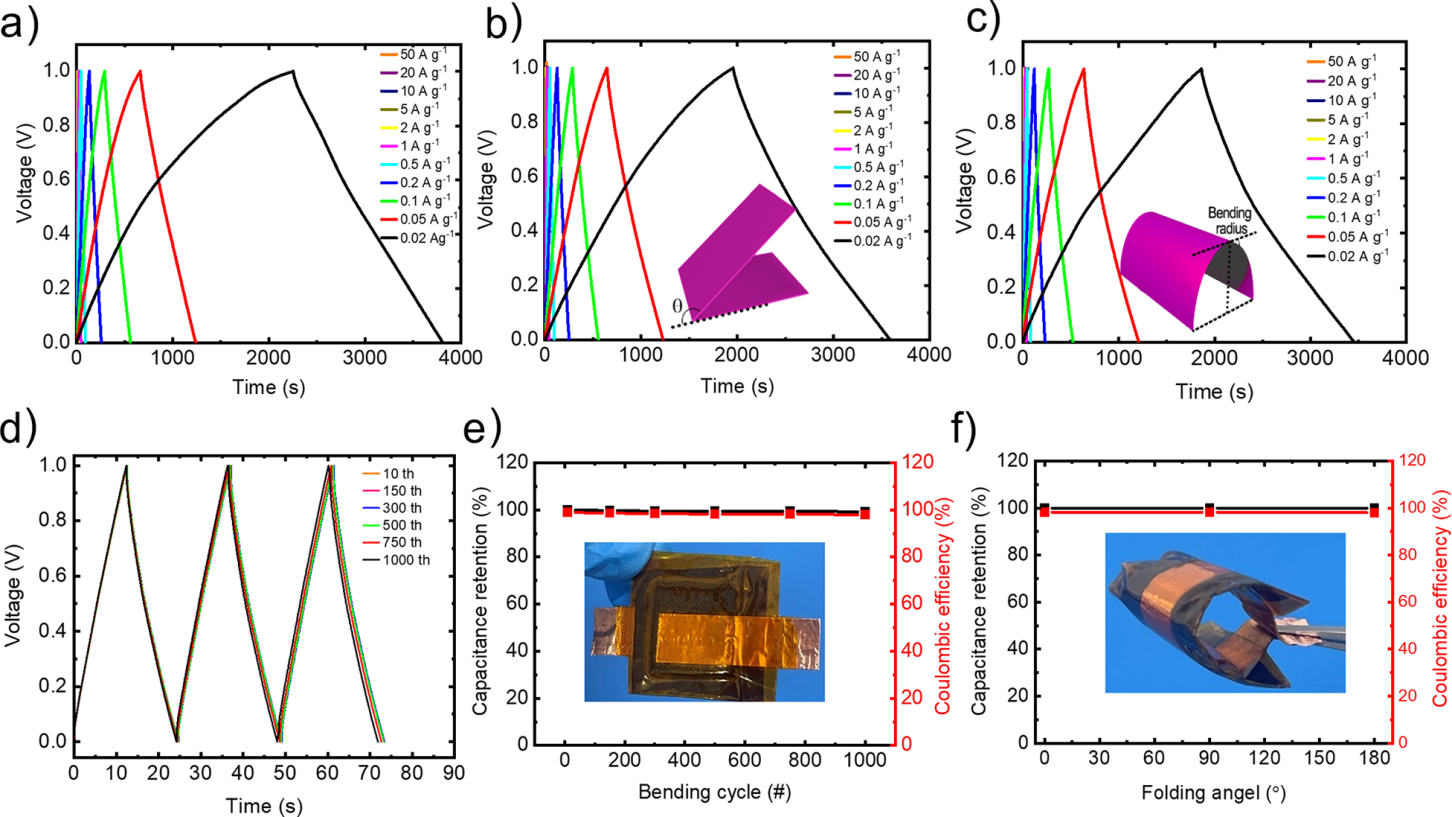
Electrochemical characterization of the prepared FSSSC. GCD curves at specific currents ranging from 0.02 to 50 A g^–1^ for (a) the FSSSC in normal state, (b) folded at 180°, and (c) after 1000 bending cycles at a curvature radius of 2 cm. (d) GCD curves of the FSSSC measured after 100, 150, 300, 500, 750, and 1000 bending cycles at 1 A g^–1^. (e) Capacitance retention and Coulombic efficiency (red, right *y*-axis) of the FSSSC over 1000 bending cycles. (f) Capacitance retention and CE (red, right *y*-axis) of the FSSSC folded at 0°, 90°, and 180°.

In particular, a FSSSC with an active area of 1.5 cm × 2 cm was produced using SPEEK-2.5%-f-NbS_2_:SPEEK composite electrolyte, sandwiched by two electrode deposited carbon cloths acting as flexible current collectors (see additional details in SI, Experimental Section). The performances of the FSSSCs were evaluated over folding until 180° and 1500 bending cycles at 10 A g^–1^. Figure 8a–c shows the GCD curve measured for a representative FSSSC at specific currents ranging from 0.02 to 50 A g^–1^ before and after 180° folding and 1000 bending cycles at a curvature radius of 2 cm. As shown by Figure 8b, the GCD curves of the FSSSC preserved their initial shapes after such bending-type stresses, proving the excellent flexibility of the device. The FSSSC also operated at a specific current as low as 0.02 A g^–1^ with a Coulombic efficiency of 70%, confirming the limited current leakage in the presence of the solid-state composite electrolyte.^162^ The prepared FSSSC exhibited a *C*_g_ of 62.3 F g^–1^ at 0.02 A g^–1^. The discrepancy between this value and the one measured for the solid-state device in rigid configuration (see Figure 6f) can be attributed to the different pressure applied to the electrodes in the rigid and flexible devices. Figure S6a reports the electrode *C*_g_ measured for the FSSSC at different specific currents, ranging from 0.02 to 50 A g^–1^. At the highest specific current of 50 A g^–1^, the FSSSC retains 61% of the *C*_g_ measured at 0.02 A g^–1^, confirming an optimal rate capability previously observed in rigid devices. In addition, Figure S6b,c shows the CV curves measured for the FSSSC folded at 180° and after 1000 bending cycles at curvature radius of 2 cm, respectively, at voltage scan rates ranging from 40 to 1500 mV s^–1^, further demonstrating the device flexibility. Figure 8d shows the comparison of the GCD curves for the SPEEK-2.5%-f-NbS_2_:SPEEK after different numbers of bending cycles (100, 150, 300, 500, 750, 1000) at 1 A g^–1^, proving that the shape of the GCD profiles of the FSSSC did not deform over subsequent mechanical stresses. This is also in accordance with the almost ideal *C*_g_ retention and high Coulombic efficiency (>98% after 1000 bending cycles) (Figure 8e). Figure 8f shows that, compared to its unbent state, the FSSSC perfectly retains its capacitance when folded at angles of 90° and 180°. The excellent stability of the FSSSC upon bending and folding is attributed to the distinctive mechanical characteristics of the optimized composite solid-state electrolyte, i.e., 2.5%-f-NbS_2_:SPEEK, as well as the to the mechanical robustness of the electrodes prepared using SPEEK as the binder and flexible carbon cloths as the current collectors.^30,126,163^

## 3 Conclusions

We have demonstrated a high-performance solid-state electrolyte based on SPEEK incorporating f-NbS_2_ nanoflakes for flexible solid-state supercapacitors (FSSSCs). The NbS_2_ nanoflakes were produced through ultrasonication-assisted liquid-phase exfoliation (LPE) of their bulk counterpart, and were then functionalized with sodium 3-mercapto-1-propanesulfonate (SMPS) salt molecules. This functionalization step is promoted by the metallicity of NbS_2_ flakes, on which −SO_3_H groups are introduced that chemically interact with the SPEEK matrix through hydrogen bonds. The nanocomposite solid-state electrolyte shows excellent mechanical, chemical, electrical, and electrochemical properties. In particular, it exhibits tensile strength up to 30.3 MPa and dimensional/chemical stabilities. The hydrogen bonding network facilitates the proton transport within the nanocomposite electrolyte through an efficient Grotthuss mechanism, improving the proton conductivity (σ) from 46.2 mS cm^–1^ in the pristine SPEEK up to 94.4 mS cm^–1^ in the optimized electrolyte (2.5%-f-NbS_2_:SPEEK). Our electrochemical characterization revealed that the effective use of our solid-state supercapacitors must be coupled with the use of ion-conducting binders (namely, SPEEK) for the electrode formulation. In fact, SPEEK can extend the ion-conducting pathways of the electrolyte in the proximity of the bound active materials, enabling a high-capacitance electrical double layer to be formed. The solid-state supercapacitors based on the optimized nanocomposite electrolyte and SPEEK as the binder reached an electrode specific (gravimetric) capacitance (*C*_g_) as high as 106 F g^–1^ at 0.05 A g^–1^, while showing optimal rate capability and cycling stability. The obtained performances outperform the ones achieved by devices based on pristine SPEEK as the solid-state electrolyte (94 F g^–1^ at 0.05 A g^–1^) or PVDF as electrically insulating binder (87 F g^–1^ at 0.05 A g^–1^). More specifically, at the high specific current of 50 A g^–1^, the use of the 2.5%-f-NbS_2_:SPEEK improved the electrode *C*_g_, energy density, and power density by 19%, 32% and 10%, respectively, compared to the SPEEK-based device (these improvements were 15%, 19%, and 1%, respectively, at the specific current of 0.02 A g^–1^). Thanks to the mechanical flexibility of our solid-state electrolytes, a FSSSC based on 2.5%-f-NbS_2_:SPEEK demonstrated an optimal capacitance retention (more than 98%) over 1000 bending cycles at a curvature radius of 2 cm and at 180° folding. Our work provides insights on the use of metallic 2D group-5 transition metal dichalcogenides for the development of advanced solid-state polymeric electrolytes for flexible electrochemical energy storage systems.

## 4 Experimental Methods

### 4.1 Materials

Poly(ether ether ketone) (PEEK) powder (Mw: 28 800 g mol^–1^), PVDF, SMPS (90%), dimethyl sulfoxide (DMSO) (≥99.9%), anhydrous isopropyl alcohol (IPA) (exfoliating solvent for the LPE of 2H/3R-NbS_2_ crystals), concentrated sulfuric acid (H_2_SO_4_, 95–98%) (sulfonating agent), and 1-methyl-2-pyrrolidone (NMP) (solvent for PEEK and SPEEK) were purchased from Sigma-Aldrich. Niobium (Nb, 99.9%, <100 μm) and sulfur (S, 99.999%, <6 mm) powders were purchased from Strem Chemicals, Inc. All the chemicals were used as received without any further purification.

### 4.2 Niobium Disulfide (NbS_2_) Crystal Production and Exfoliation

NbS_2_ nanoflakes were produced through ultrasonication-assisted LPE of bulk 2H/3R-NbS_2_ crystals, synthesized through the direct reaction from Nb and S elements, as described in previous studies.^104^ See the Supporting Information for experimental details.

### 4.3 Functionalization of NbS_2_ Nanoflakes

See the Supporting Information for experimental details.

### 4.4 PEEK Sulfonation

The sulfonation of PEEK powder was carried via a direct sulfonation reaction with an optimum (in terms of corresponding σ) DS of 70.2%, in agreement with our previous studies.^75^ See the Supporting Information for experimental details.

### 4.5 Electrode and Electrolyte Preparation and Solid-State Supercapacitor Assembly

As shown in Figure 1b, flexible electrodes were prepared using activated carbon powder (AB520Y, MTI corporation) (72 wt %) mixed with single/few-layer graphene (BeDimensional S.p.A.) (8 wt %) as the active material, and carbon black (Super-P, Alfa Aesar) (10 wt %) as the conductive additive. See the Supporting Information for further experimental details.

### 4.6 Material and Device Characterization

See the Supporting Information for experimental details.

## Supporting Information Available

Experimental section; Supporting information and device characterizations (SEM, TEM, AFM, WU, MS, σ, TGA, strain–stress curve, CV, and GCD measurements) (PDF)
