# Supplementary material for: Functionalized Metallic 2D Transition Metal Dichalcogenide-Based Solid-State Electrolyte for Flexible All-Solid-State Supercapacitors
Source: ACS Nano. 2022 Oct 4;16(10):16426–42. doi: 10.1021/acsnano.2c05640 (PMC9620411; doi:10.1021/acsnano.2c05640)
Supplement: Supplementary file 1 — nn2c05640_si_001.pdf [file nn2c05640_si_001.pdf]

Supplementary Information

Functionalized metallic 2D transition metal dichalcogenide-based solid-state electrolyte for flexible all-solid-state supercapacitors

Ahmad Bagheri,[a,b,‡] *Sebastiano Bellani,*[c,‡,*] *Hossein Beydaghi,*[c] *Matilde Eredia,*[c] *Leyla Najafi,*[c] *Gabriele Bianca,*[a,d] *Marilena Isabella Zappia,*[c] *Milad Safarpour,*[e,f] *Maedeh Najafi,*[e,f] *Elisa Mantero,*[c] *Zdenek Sofer,*[g] *Guorong Hou,*[g] *Vittorio Pellegrini,* [a,c] *Xinliang Feng,*[b,h] *and Francesco Bonaccorso*[a,c,*]

[a] Graphene Labs, Istituto Italiano di Tecnologia, via Morego 30, 16163 Genoa, Italy

[b] Center for Advancing Electronics Dresden (cfaed) & Faculty of Chemistry and Food Chemistry, Technische Universität Dresden, 01062 Dresden, Germany

[c] BeDimensional SpA, Lungotorrente Secca 30R, 16163 Genoa, Italy

* E-mail: francesco.bonaccorso@iit.it, s.bellani@bedimensional.it

[d] Dipartimento di Chimica e Chimica Industriale, Università degli Studi di Genova, via Dodecaneso 31, 16146 Genoa, Italy

[e] Smart Materials, Istituto Italiano di Tecnologia, Via Morego 30, 16163-Genova, Italy

[f] Dipartimento di Informatica Bioingegneria, Robotica e Ingegneria dei Sistemi (DIBRIS), Universita Degli Studi di Genova, Via All'Opera Pia 13, 16145 Genova, Italy

[g] Department of Inorganic Chemistry, University of Chemistry and Technology Prague, Technicka 5, 166 28 Prague 6, Czech Republic

[h] Max Planck Institute of Microstructure Physics, Weinberg 2, 06120 Halle, Germany

‡ These authors contributed equally

EXPERIMENTAL SECTION

Niobium disulphide (NbS_2) crystal production and exfoliation. NbS_2 nanoflakes were produced through ultrasonication-assisted LPE of bulk 2H/3R-NbS_2 crystals, synthesized through the direct reaction from Nb and S elements, as described in previous studies[1,2]. Experimentally, powders of Nb and S powders at a nearly 1:2 elemental stoichiometry (1 wt% excess of S to avoid Nb-

* E-mail: francesco.bonaccorso@iit.it, s.bellani@bedimensional.it

intercalated compounds)[3] were inserted in a quartz glass ampoule. The loaded powders were heated with a three-step protocol at 450 ˚C for 12 h, at 600 ˚C for 48 h, at 900 ˚C for 48 h, and then cooled down to room temperature. The 50 mg of the as-produced crystals were added to 50 mL of IPA. The so-obtained dispersion was ultrasonicated for 6 h using a sonicator (Branson® 5800 cleaner, Branson Ultrasonics). Subsequently, the dispersion was ultracentrifuged using a Beckman Coulter centrifuge (Optima™ XE-90 with an SW32Ti rotor) at 2700 g for 20 min at 15 ˚C to separate the exfoliated material (nanoflakes) in the supernatant from the sediment consisting of unexfoliated material. Then, 80% of the supernatant was collected to obtain a dispersion of NbS_2 nanoflakes with a concentration of 0.91 g L^{-1}, as measured through gravimetric method. Commercial crystals of 2H-MoS_2 (HQ Graphene) were also exfoliated according to the above procedure.

Functionalization of NbS_2 nanoflakes. As sketched in **Figure 1a** of the main text, 10 mL of the as-produced NbS_2 nanoflakes dispersion (10 g L^{-1}) were mixed with 410 mg of SMPS (dispersed in 10 mL of DMSO) and heated at 70 ˚C for 5 h under magnetic stirring. The excess of SMPS was removed by recovering the functionalized material through 10 min of centrifugation at 2599 g (Sigma 3-16P centrifuge, rotor 19776), followed by two subsequent washing steps with a IPA:

* E-mail: francesco.bonaccorso@iit.it, s.bellani@bedimensional.it

DMSO (1.5:1 vol%) and IPA, respectively, with the assistance of ultracentrifugation for 20 min at 106800 g (Sigma 3-16P centrifuge, rotor 19776). Finally, the so-produced functionalized NbS_2 (f-NbS_2) nanoflakes were redispersed in NMP by ultrasonication for 5 min to obtain the final mixture concentration of 18 mg mL^{-1}. 2H-MoS_2 nanoflakes were also functionalized according to the above procedure.

PEEK sulfonation. The sulfonation of PEEK powder was carried *via* direct sulfonation reaction with an optimum (in terms of corresponding σ) degree of sulfonation (DS) of 70.2 %, in agreement with our previous studies[4]. Briefly, 1 g of PEEK powder was dried at 70 ˚C for 1 h and then added to 10 mL of concentrated H_2SO_4 (95–98%). The resulting reaction mixture was vigorously stirred for 1 h. After completely dissolving the PEEK powder, the mixture was heated at 60 ˚C and mixed for 5 h and 20 min. The obtained mixture was cooled down to 25 ˚C and added dropwise to a large amount of ice-water to precipitate the SPEEK. The obtained material was washed several times with deionized water until reaching a neutral pH (~ 7). Afterward, the material was collected by filtering, and dried at 70 ˚C overnight to obtain SPEEK polymer.

Electrode and electrolyte preparation and solid-state supercapacitor assembly. As shown in **Figure 1b** of the main text, flexible electrodes were prepared using activated carbon powder

* E-mail: francesco.bonaccorso@iit.it, s.bellani@bedimensional.it

(AB520Y, MTI corporation) (72 wt%) mixed with single-/few-layer graphene (BeDimensional S.p.A.) (8 wt%) as the active material, and carbon black (Super-P, Alfa Aesar) (10 wt%) as the conductive additive. Single-/few-layer graphene was produced through wet-jet milling exfoliation,[5,6] and the formulation of the active material (*i.e.,* activated carbon:single-/few-layer graphene weight ratio of 90:10) followed the protocols reported in our previous works.[7,8] Also, SPEEK and PVDF polymers were used with a 10 wt% content as ion-conducting and electrically insulating binders, respectively. To prepare a homogeneous slurry, the polymers (PVFD and SPEEK) were first dissolved in NMP at 10 wt%. Then, activated carbon and super-P were mixed with the binder dissolved in NMP using a planetary centrifugal mixer (Thinky, ARE 200, USA). The slurry was stirred for 12 h at room temperature and then deposited onto carbon cloths by Dr. blading. The resulting electrodes were placed in a vacuum oven (VD 56, Binder) at 80 ˚C for 24 h to remove solvent residuals. The mass of the electrode materials deposited on carbon cloth was 1.4 mg cm^2. **Figure 1c** of the main text illustrates the fabrication of the solid-state nanocomposite electrolytes made of f-NbS_2-incorporating SPEEK. A series of nanocomposite electrolytes (x-f-NbS_2:SPEEK, in which x is the weight percentage of the f-NbS_2 nanoflakes) were synthesized *via* solution casting method by varying the mass ratios of the f-NbS_2 nanoflakes. Experimentally, a

* E-mail: francesco.bonaccorso@iit.it, s.bellani@bedimensional.it

solution comprising SPEEK and NMP (1:10 wt/vol) was stirred at 60 ˚C to dissolve the polymer and obtain a homogeneous solution. Afterward, various weight percentages of f-NbS_2 nanoflakes dispersed in NMP were added to the SPEEK solution, keeping the mixture stirred for 4 h at 60 ˚C. Then, the nanocomposite electrolytes were produced by casting method using a Dr. blade. The as-prepared films were dried at 80 ˚C overnight, at 120 ˚C for 12 h, and 140 ˚C for 4 h. Also, the pristine SPEEK electrolyte was prepared in a similar way to the nanocomposite ones but without adding the f-NbS_2 nanoflakes. After drying, the solid-state electrolyte thickness was 80 μm, as measured through contact profilometry (XP2, Ambios Technology). The dried electrolytes were activated with 1 M H_2SO_4 and then washed with ultrapure water. Solid-state electrolytes based on f-MoS_2 nanoflakes instead of f-NbS_2 ones were also produced for comparison, following the same fabrication procedures. The resulting solid-state electrolytes were used in form of self-standing membranes to fabricate solid-state supercapacitors. Rigid configurations were produced by sandwiching the solid-state electrolytes between two electrodes using a Swagelok cell. **Table S1** lists all the investigated devices. Noteworthy, a traditional electrochemical double layer capacitor (EDLC) using PVDF as the binder, 1 M H_2SO_4 as the liquid (aqueous) electrolyte, and a glass fiber as separator (Whatman) (device named PVDF-1 M H_2SO_4) was also produced and

* E-mail: francesco.bonaccorso@iit.it, s.bellani@bedimensional.it

characterized as aqueous EDLC for comparison. After the characterization of these rigid supercapacitor configurations, a flexible solid-state supercapacitor (FSSSC) based on the most performant combination of solid-state electrolyte and electrode binder was produced by sandwiching the solid-state electrolyte between two electrodes and copper tape and applying a pressure of 1 MPa. The resulting device was packed using a 0.06 mm-thick Kapton tape.

Table S1. List of the investigated solid-state supercapacitors and the reference EDLC using 1 M H_2SO_4 as the liquid (aqueous) electrolyte.

**Samples name**	**Electrode**	**Binder**	**Electrolyte**
PVDF-1M H_2SO_4 (Ref.)	Activated carbon + Carbon black	PVDF	1M H_2SO_4
PVDF-SPEEK	Activated carbon + Carbon black	PVDF	SPEEK
SPEEK-SPEEK	Activated carbon + Carbon black	SPEEK	SPEEK
SPEEK-f-NbS_2:SPEEK	Activated carbon + Carbon black	SPEEK	f-NbS_2:SPEEK

Material and device characterization. Transmission electron microscopy (TEM) images of the exfoliated materials were acquired using a JEM 1011 (JEOL) transmission electron microscope

* E-mail: francesco.bonaccorso@iit.it, s.bellani@bedimensional.it

(thermionic W filament), operated with an acceleration voltage of 100 kV. The samples were prepared by depositing diluted dispersions of the exfoliated materials onto an ultrathin C-film on holey carbon 400 mesh Cu grids (Ted Pella Inc) by drop casting. The samples were subsequently dried under vacuum at room temperature. The morphological and statistical analyses of the lateral dimension of the produced nanoflakes were carried out using ImageJ software (NIH) and OriginPro 9.1 software (OriginLab), respectively. Atomic force microscopy (AFM) images of the exfoliated materials were acquired using a Bruker Dimension Icon atomic force microscope (Bruker Dimension Icon, Billerica, MA, USA). The measurements were carried out in intermittent contact mode using RTESPA cantilevers (Bruker, Billerica, MA, USA) with a tip with a nominal diameter of 8 nm. A driving frequency of ~300 kHz was used for the image acquisition. The images were collected over an area of 2.5×2.5 μm^2 (512×512 data points), using a scan rate of 0.7 Hz. The samples were prepared by depositing the diluted dispersions of the exfoliated materials onto mica substrates (G250-1, Agar Scientific Ltd.). The height profile analysis was performed using Gwydion 2.54 software. The statistical analysis of the AFM data was performed using OriginPro 9.1 software. X-ray diffraction (XRD) analysis of the exfoliated materials was performed using a PANalytical Empyrean X-ray diffractometer with Cu Kα

* E-mail: francesco.bonaccorso@iit.it, s.bellani@bedimensional.it

radiation, while a Renishaw micro-Raman Invia 1000 spectrophotometer was used for the Raman spectroscopy analysis at 532 nm exciting wavelength. For both XRD and Raman analyses, the samples were prepared by drop casting diluted dispersion of the exfoliated materials onto the Si/SiO_2 substrates and dried under vacuum for 12 h. Scanning electron microscopy (SEM) and energy-dispersive X-ray spectroscopy (EDX) analyses were carried out using a scanning electron microscope (SEM, JEOL JSM-6490LA SEM Analytical microscope). Fourier-transform infrared (FTIR) spectroscopy measurements of the produced nanocomposite solid-state electrolytes were carried out using an Equinox 70 FTIR spectrometer coupled with the attenuated total reflection (ATR) accessory (MIRacle ATR, Pike Technologies), scanning wavenumbers from 600 to 3800 cm^{-1} at room temperature.

The water uptake (WU) and membrane swelling (MS) of the solid-state electrolytes were calculated using the following method. First, a cut piece of the produced electrolyte was dried overnight at 90 ˚C, and then its weight (W_d) and area (A_d) were measured. Afterward, the solid-state electrolyte was immersed in deionized water for 12 h at room temperature and after blot-drying the residual water with two clean filter papers, the weight (W_w) and area (A_w) of the

* E-mail: francesco.bonaccorso@iit.it, s.bellani@bedimensional.it

solid-state electrolyte were immediately measured. Finally, the WU and MS of the solid-state electrolyte were calculated using the following equations:

$$WU\,(\%) = \frac{W_w - W_d}{W_d} \times 100 \quad (1)$$

$$MS\,(\%) = \frac{A_w - A_d}{A_d} \times 100 \quad (2)$$

The proton conductivity (σ) of the solid-state electrolytes was measured through electrochemical impedance spectroscopy (EIS) measurements using two platinum electrodes over a frequency range of 2 mHz–100 kHz with an AC voltage amplitude of 0.05 V. The samples were prepared by soaking the as-produced solid-state electrolytes in deionized water for 24 h. The σ of the produced electrolytes was determined by:

$$\sigma = \frac{L}{R.A} \quad (3)$$

where L is the electrolyte thickness (cm), A is the electrolyte area (cm^2) and R is defined as the electrolyte resistance (Ω) obtained from the Nyquist plots (*i.e.*, real part of the impedance at high frequency > 10 kHz). The tensile tests were performed using a dual column Instron 3365 universal testing machine equipped with a 10 N cell load and pneumatic clamps. Dumbbell-shaped ISO 527-2 type 5A samples with a thickness of 1 mm were cut out directly from prepared

* E-mail: francesco.bonaccorso@iit.it, s.bellani@bedimensional.it

solid-state electrolytes. Each sample was measured three times, averaging the extrapolated characteristics. Strain displacement was applied with rates ranging 0.2 mm min^{-1} at 25 ˚C. Thermogravimetric analysis (TGA) was performed to investigate the thermal stability of the produced solid-state electrolytes using a TGA Q500 (TA Instruments, USA) thermogravimetric analyzer in N_2 atmosphere from 25 to 800 ˚C at a heating rate of 10 ˚C min^{-1}. The electrochemical characterization of the supercapacitors included cyclic voltammetry (CV), galvanostatic charge/discharge (GCD), and EIS measurements, which were performed using a potentiostat/galvanostat (VMP3, Biologic) at room temperature (25 ˚C). Cyclic voltammetry measurements were carried out at voltage scan rates ranging from 5 mV s^{-1} to 1500 V s^{-1}. Galvanostatic charge/discharge measurements were carried out at different specific currents, ranging from 0.02 to 50 A g^{-1}. The specific (gravimetric) capacitance (C_g) of the electrode was calculated according to the following equations:

$$C_g = \frac{\int i dV}{ms\Delta V} \quad \text{from CV curves} \qquad (4)$$

$$C_g = 2 \times \frac{|i|}{m} \times \frac{t_d}{\Delta V} \quad \text{from GCD curves} \qquad (5)$$

* E-mail: francesco.bonaccorso@iit.it, s.bellani@bedimensional.it

where ΔV is the voltage cell window, $\int i dV$ is the integrated area of the CV curve, m is the mass loading of the electrode (excluding the current collector), s is the scan rate, V is the cell voltage, i is the charging/discharging current, t_d is the discharge time of the GCD curve.

The energy density and power density of the supercapacitors were calculated by:

$$\text{energy density (Wh kg}^{-1}) = \frac{i_d}{3.6}\int V dt \quad (6)$$

$$\text{power density (W kg}^{-1}) = 3600 \times \frac{E}{t_d} \quad (7)$$

in which $\int V dt$ is the area under the galvanostatic discharge curve and i_d is the specific current (A g^{-1}). Electrochemical impedance spectroscopy measurements of the supercapacitors were acquired in the 0.01 Hz–200 kHz frequency range at discharged state with an AC voltage amplitude of 0.02 V.

* E-mail: francesco.bonaccorso@iit.it, s.bellani@bedimensional.it

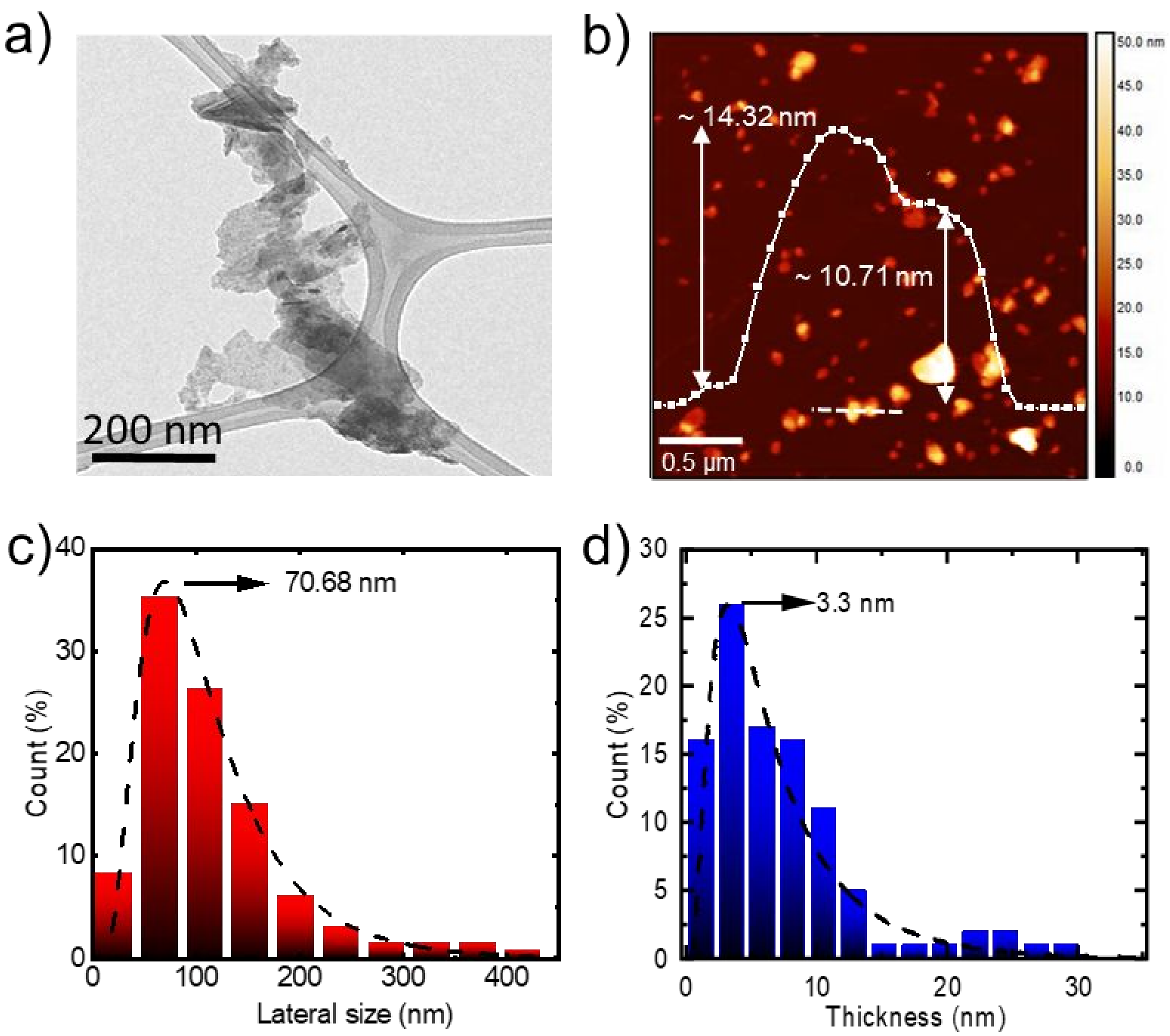

Figure S1. (a) TEM and (b) AFM images of representative pristine NbS_2 nanoflakes. (c) Lateral size and (d) thickness statistical analyses for the pristine NbS_2 nanoflakes.

* E-mail: francesco.bonaccorso@iit.it, s.bellani@bedimensional.it

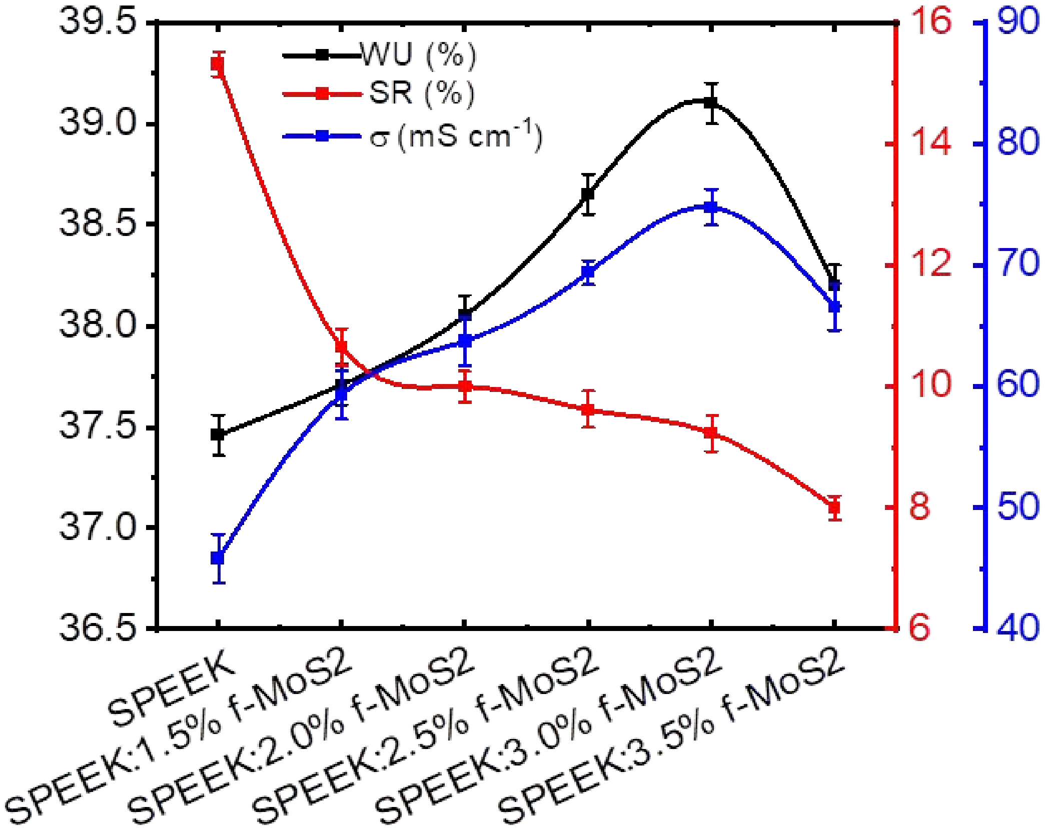

Figure S2. WU, MS, and σ of the prepared solid-state electrolytes based on f-MoS_2 nanoflakes. These properties were compared with those obtained by the composite solid-state electrolytes based on f-NbS_2 nanoflakes, as shown in **Figure 4f** of the main text.

* E-mail: francesco.bonaccorso@iit.it, s.bellani@bedimensional.it

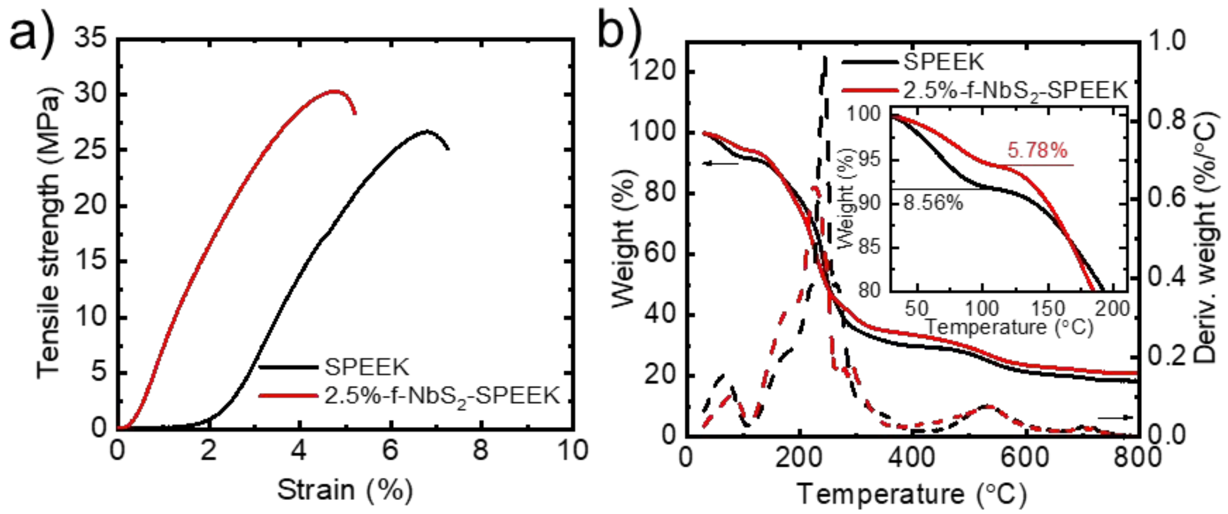

Figure S3. (a) Strain-stress curves (b) TGA curves of the SPEEK and the 2.5%-f-NbS_2:SPEEK electrolytes. The inset panel shows the TGA curve in the 30-210 ˚C temperature range.

Table S2. Mechanical properties of the SPEEK and 2.5%-f-NbS_2:SPEEK electrolytes at room temperature.

Samples	Tensile strength [MPa]	Young's Modulus [MPa]	Elongation at break (%)
SPEEK	26.7	826.4	7.3
2.5%-f-NbS_2:SPEEK	30.3	1066.3	5.2

* E-mail: francesco.bonaccorso@iit.it, s.bellani@bedimensional.it

Figure S4a and **S4b** show the top-view and cross-sectional SEM images of a representative supercapacitor electrode based on SPEEK as the binder and using carbon cloth as the current collector. The carbon cloth is made of interconnected carbon fibers that are coated by the other electrode materials (*i.e.*, activated carbon, single-/few-layer graphene and SPEEK).

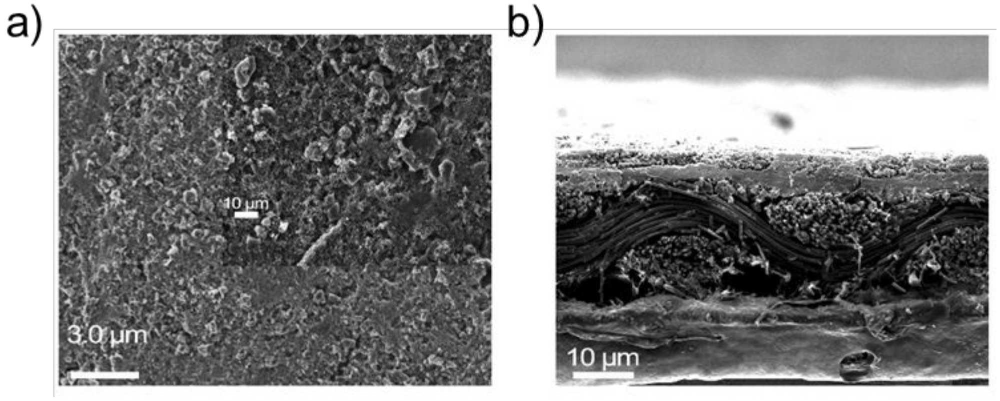

Figure S4. (a) Top-view and (b) cross-sectional SEM images of a representative supercapacitor electrode based on SPEEK as the binder and carbon cloth as the current collector.

* E-mail: francesco.bonaccorso@iit.it, s.bellani@bedimensional.it

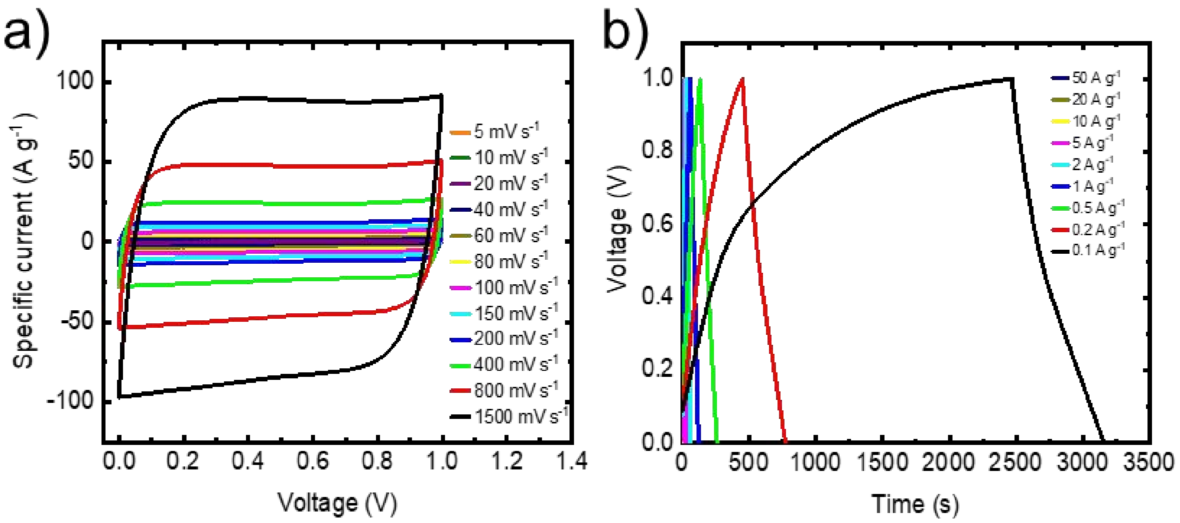

Figure S5. Electrochemical characterization of the investigated 1 M H_2SO_4-based EDLC reference. (a) CV curves acquired at voltage scan rates ranging from 5 to 1500 mV s^{-1}.(b) GCD curves acquired at specific currents ranging from 0.1 to 50 A g^{-1}.

Table S3. Comparison between the capacitance, energy density and power density measured for SPEEK-2.5% -f-NbS_2:SPEEK and those reported in literatures for relevant FSSSCs.

S. N.	Electrode	Electrolyte	Potential windows (V)	Capacitance (F g^{-1})	Energy density (W h Kg^{-1})	Power density (kW kg^{-1})	Cyclic stability	Ref.

* E-mail: francesco.bonaccorso@iit.it, s.bellani@bedimensional.it

1	AC	polyelectrolyte-based gel polymer electrolyte	1.2	64.92	13.26	2.26	94.63% after 10000 cycles	[9]
2	AC film	carboxylated chitosan hydrogel	1	45.9	5.2	0.2266	-	[10]
3	activated reduced graphene oxide	H_2SO_4−PVA	0.8	53	4.7	0.402	-	[11]
4	N/S co-doped hierarchical porous carbons (NS-HPCS)	PVA/KOH	1.5	50	8.01	0.4	94.5% after 5000 cycles	[12]
5	nitrogen-doped graphene nanosheet	PVA–$LiClO_4$ with 10 wt% phloroglucinol	1.2	114	2.3	0.150	94% after 5000 cycles	[13]
6	AC	PVA/PVP/EMIHSO$_4$/HQ	1.2	188	9.0	4.2		[14]

* E-mail: francesco.bonaccorso@iit.it, s.bellani@bedimensional.it

AC	2.5%-f-NbS_2:SPEEK	1	116	7.2	20.1	99.1% after 30000 cycles	**This work**

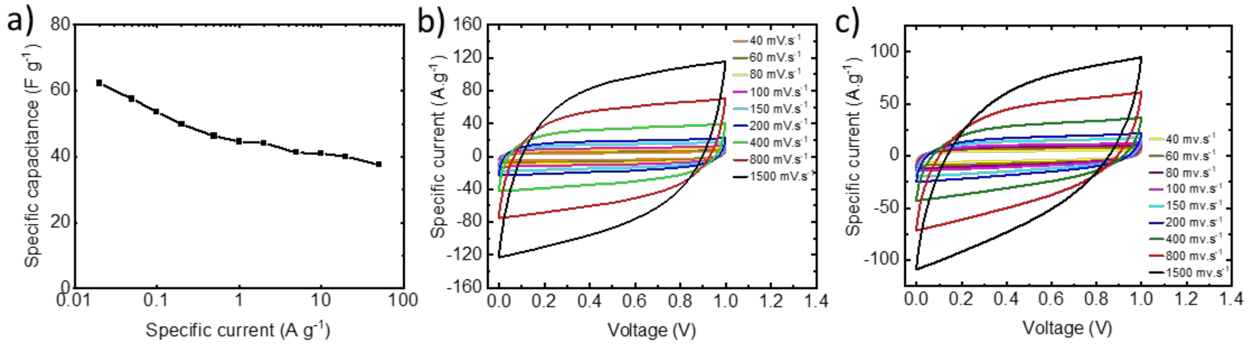

Figure S6. Electrochemical characterization of the FSSSC. (a) Specific capacitance *vs.* specific current plot measured for the SPEEK-2.5%-f-NbS_2:SPEEK-based FSSSC in normal state. (b) CV curves measured for the FSSSC folded at 180 ^{0} and (c) after 1000 bending cycles at a curvature radius of 2 cm, at voltage scan rates ranging from 40 to 1500 mV s^{-1}.

REFERENCES

(1) Najafi, L.; Bellani, S.; Oropesa-Nuñez, R.; Martín-García, B.; Prato, M.; Mazánek, V.; Debellis, D.; Lauciello, S.; Brescia, R.; Sofer, Z.; Bonaccorso, F. Niobium Disulphide (NbS_2)-Based (Heterogeneous) Electrocatalysts for an Efficient Hydrogen Evolution Reaction. *J. Mater. Chem. A* **2019**, *7* (44), 25593–25608.

* E-mail: francesco.bonaccorso@iit.it, s.bellani@bedimensional.it

(2) Chia, X.; Ambrosi, A.; Lazar, P.; Sofer, Z.; Pumera, M. Electrocatalysis of Layered Group 5 Metallic Transition Metal Dichalcogenides (MX_2, M = V, Nb, and Ta; X = S, Se, and Te). *J. Mater. Chem. A* **2016**, *4* (37), 14241–14253.

(3) Leroux, M.; Cario, L.; Bosak, A.; Rodière, P. Traces of Charge Density Waves in NbS_2. *Phys. Rev. B* **2018**, *97* (19), 1–6.

(4) Bagheri, A.; Javanbakht, M.; Beydaghi, H.; Salarizadeh, P.; Shabanikia, A.; Amoli, and H. S.; Salar Amoli, H. Sulfonated Poly(Etheretherketone) and Sulfonated Polyvinylidene Fluoride-Co-Hexafluoropropylene Based Blend Proton Exchange Membranes for Direct Methanol Fuel Cell Applications. *RSC Adv.* **2016**, *6* (45), 39500–39510.

(5) Del Rio Castillo, A. E.; Pellegrini, V.; Ansaldo, A.; Ricciardella, F.; Sun, H.; Marasco, L.; Buha, J.; Dang, Z.; Gagliani, L.; Lago, E.; Curreli, N.; Gentiluomo, S.; Palazon, F.; Prato, M.; Oropesa-Nuñez, R.; Toth, P. S.; Mantero, E.; Crugliano, M.; Gamucci, A.; Tomadin, A.; Polini, M.; Bonaccorso, F. High-Yield Production of 2D Crystals by Wet-Jet Milling. *Mater. Horizons* **2018**, *5* (5), 890–904.

(6) Bellani, S.; Petroni, E.; Del Rio Castillo, A. E.; Curreli, N.; Martín-García, B.; Oropesa-Nuñez, R.; Prato, M.; Bonaccorso, F. Scalable Production of Graphene Inks via Wet-Jet

* E-mail: francesco.bonaccorso@iit.it, s.bellani@bedimensional.it

Milling Exfoliation for Screen-Printed Micro-Supercapacitors. *Adv. Funct. Mater.* **2019**, *29* (14), 1–14.

(7) Bellani, S.; Martín-Garciá, B.; Oropesa-Nuñez, R.; Romano, V.; Najafi, L.; Demirci, C.; Prato, M.; Del Rio Castillo, A. E.; Marasco, L.; Mantero, E.; D'Angelo, G.; Bonaccorso, F. "ion Sliding" on Graphene: A Novel Concept to Boost Supercapacitor Performance. *Nanoscale Horizons* **2019**, *4* (5), 1077–1091.

(8) Garakani, M. A.; Bellani, S.; Pellegrini, V.; Oropesa-Nuñez, R.; Castillo, A. E. D. R.; Abouali, S.; Najafi, L.; Martín-García, B.; Ansaldo, A.; Bondavalli, P.; Demirci, C.; Romano, V.; Mantero, E.; Marasco, L.; Prato, M.; Bracciale, G.; Bonaccorso, F. Scalable Spray-Coated Graphene-Based Electrodes for High-Power Electrochemical Double-Layer Capacitors Operating over a Wide Range of Temperature. *Energy Storage Mater.* **2021**, *34*, 1–11.

(9) Yan, C.; Jin, M.; Pan, X.; Ma, L.; Ma, X. A Flexible Polyelectrolyte-Based Gel Polymer Electrolyte for High-Performance All-Solid-State Supercapacitor Application. *RSC Adv.* **2020**, *10* (16), 9299–9308.

(10) Yang, H.; Liu, Y.; Kong, L.; Kang, L.; Ran, F. Biopolymer-Based Carboxylated Chitosan

* E-mail: francesco.bonaccorso@iit.it, s.bellani@bedimensional.it

Hydrogel Film Crosslinked by HCl as Gel Polymer Electrolyte for All-Solid-Sate Supercapacitors. *J. Power Sources* 2019, *426*, 47–54.

(11) Chang, H. W.; Lu, Y. R.; Chen, J. L.; Chen, C. L.; Chen, J. M.; Tsai, Y. C.; Chou, W. C.; Dong, C. L. Electrochemically Activated Reduced Graphene Oxide Used as Solid-State Symmetric Supercapacitor: An X-Ray Absorption Spectroscopic Investigation. *J. Phys. Chem. C* 2016, *120* (39), 22134–22141.

(12) Huo, S.; Liu, M.; Wu, L.; Liu, M.; Xu, M.; Ni, W.; Yan, Y. M. Methanesulfonic Acid-Assisted Synthesis of N/S Co-Doped Hierarchically Porous Carbon for High Performance Supercapacitors. *J. Power Sources* 2018, *387*, 81–90.

(13) Le, P. A.; Nguyen, V. T.; Yen, P. J.; Tseng, T. Y.; Wei, K. H. A New Redox Phloroglucinol Additive Incorporated Gel Polymer Electrolyte for Flexible Symmetrical Solid-State Supercapacitors. *Sustain. Energy Fuels* 2019, *3* (6), 1536–1544.

(14) Yadav, N.; Yadav, N.; Hashmi, S. A. Ionic Liquid Incorporated, Redox-Active Blend Polymer Electrolyte for High Energy Density Quasi-Solid-State Carbon Supercapacitor. *J. Power Sources* 2020, *451*, 227771.

* E-mail: francesco.bonaccorso@iit.it, s.bellani@bedimensional.it

* E-mail: francesco.bonaccorso@iit.it, s.bellani@bedimensional.it
